# Self‐Reinforced Bimetallic Mito‐Jammer for Ca^2+^ Overload‐Mediated Cascade Mitochondrial Damage for Cancer Cuproptosis Sensitization

**DOI:** 10.1002/advs.202306031

**Published:** 2024-02-11

**Authors:** Chier Du, Xun Guo, Xiaoling Qiu, Weixi Jiang, Xiaoting Wang, Hongjin An, Jingxue Wang, Yuanli Luo, Qianying Du, Ruoyao Wang, Chen Cheng, Yuan Guo, Hua Teng, Haitao Ran, Zhigang Wang, Pan Li, Zhiyi Zhou, Jianli Ren

**Affiliations:** ^1^ Department of Ultrasound and Chongqing Key Laboratory of Ultrasound Molecular Imaging the Second Affiliated Hospital of Chongqing Medical University Chongqing 400010 P. R. China; ^2^ Department of Intensive Care Unit the Second Affiliated Hospital of Chongqing Medical University Chongqing 400010 P. R. China; ^3^ Department of Radiology Second Affiliated Hospital of Chongqing Medical University Chongqing 400010 P. R. China; ^4^ Department of Breast and Thyroid Surgery Second Affiliated Hospital of Chongqing Medical University Chongqing 400010 P. R. China; ^5^ Department of General Practice Chongqing General Hospital Chongqing 400010 P. R. China

**Keywords:** Ca^2+^ overload, cascade mitochondria damage, chemodynamic therapy, cuproptosis, immunotherapy

## Abstract

Overproduction of reactive oxygen species (ROS), metal ion accumulation, and tricarboxylic acid cycle collapse are crucial factors in mitochondria‐mediated cell death. However, the highly adaptive nature and damage‐repair capabilities of malignant tumors strongly limit the efficacy of treatments based on a single treatment mode. To address this challenge, a self‐reinforced bimetallic Mito‐Jammer is developed by incorporating doxorubicin (DOX) and calcium peroxide (CaO_2_) into hyaluronic acid (HA) ‐modified metal‐organic frameworks (MOF). After cellular, Mito‐Jammer dissociates into CaO_2_ and Cu^2+^ in the tumor microenvironment. The exposed CaO_2_ further yields hydrogen peroxide (H_2_O_2_) and Ca^2+^ in a weakly acidic environment to strengthen the Cu^2+^‐based Fenton‐like reaction. Furthermore, the combination of chemodynamic therapy and Ca^2+^ overload exacerbates ROS storms and mitochondrial damage, resulting in the downregulation of intracellular adenosine triphosphate (ATP) levels and blocking of Cu‐ATPase to sensitize cuproptosis. This multilevel interaction strategy also activates robust immunogenic cell death and suppresses tumor metastasis simultaneously. This study presents a multivariate model for revolutionizing mitochondria damage, relying on the continuous retention of bimetallic ions to boost cuproptosis/immunotherapy in cancer.

## Graphical abstract

Self‐reinforced bimetallic Mito‐Jammer is rationally designed and synthesized by loading DOX and CaO_2_ into the hyaluronic acid‐modified metal‐organic framework‐199. By triggering reactive oxygen species storm and Ca^2+^ overload, the Mito‐Jammer could enhance the tumor‐specific Cu^2+^ aggregation and induce cascade mitochondrial damage to further arouse tumor‐specific cuproptosis and cuproptosis‐related immunotherapy.

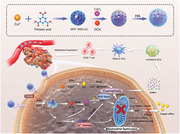

## 1 Introduction

The survival and proliferation of cancer cells rely heavily on the homeostasis of subcellular organelles.^[^ ^1^, ^2^ ^]^ These organelles participate in signaling pathways that drive malignant behavior including heightened metabolism^[^ ^3^, ^4^ ^]^ and anti‐oxidative stress responses.^[^ ^5^, ^6^ ^]^ Current approaches, such as chemotherapy,^[^ ^7^, ^8^, ^9^ ^]^ gene therapy,^[^ ^10^, ^11^ ^]^ and reactive oxygen species (ROS)‐based therapies,^[^ ^12^, ^13^, ^14^, ^15^ ^]^ are commonly used to regulate cellular organelles due to their action sites within the organelles. However, the nonspecific diffusion of drugs can disrupt normal cellular ecology, necessitating the development of personalized strategies for the precise intracellular regulation of subcellular organelles.^[^ ^16^, ^17^ ^]^

In recent years, numerous nanomedicines have been designed to target specific subcellular organelles such as mitochondria, lysosomes, and the endoplasmic reticulum.^[^ ^18^, ^19^ ^]^ However, these nanotechnologies, which predominantly rely on a single treatment mode, such as photothermal therapy (PTT) or sonodynamic therapy (SDT), often fail to induce sustained organelle injury.^[^ ^20^ ^]^ This limited efficacy can be attributed to the remarkable adaptive properties and damage repair capabilities of malignant tumors.^[^ ^21^ ^]^ Mitochondria, which serve as the primary intracellular energy source, play critical roles in regulating redox balance, the cellular tricarboxylic acid (TCA) cycle, and apoptosis in eukaryotic cells.^[^ ^22^, ^23^ ^]^ Furthermore, mitochondria act as the central hub for metal ion metabolism, and their destruction exacerbates ion oxidative stress for cell death.^[^ ^24^, ^25^ ^]^ Consequently, a multidimensional approach that targets mitochondria to amplify interference with ion regulation represents an ideal strategy for antitumor interventions.^[^ ^26^ ^]^

Chemodynamic therapy (CDT) is an effective antitumor strategy, where Fenton/Fenton‐like ions, such as Cu^2+^, Fe^3+^, and Mn^2+^, can significantly enhance ROS accumulation in tumor cells, thus interfering with mitochondrial physiological activity.^[^ ^27^, ^28^ ^]^ In addition, a promising copper‐dependent death pathway, termed “cuproptosis,” has been identified recently.^[^ ^29^ ^]^ In contrast to other known forms of cell death, cuproptosis involves an abnormal TCA cycle, which ultimately leads to proteotoxic stress.^[^ ^30^, ^31^ ^]^ Thus, the exogenous introduction of copper is a good candidate for exerting CDT and cuproptosis simultaneously, and this analog cuproptosis‐based synergistic cancer therapy has become the main focus of current nanomedicine research. Small‐molecule drugs, ultrasound, PTT, and other technologies have been used to enhance cuproptosis.^[^ ^32^, ^33^ ^]^ However, the high expression of the copper transporter family, including copper transporter and copper transport phosphorylating ATPase (Cu‐ATPase), in tumor cells may prevent cuproptosis, which transfers copper into the extracellular space through ATPase hydrolysis. Therefore, reducing the copper efflux is a formidable challenge for us to sensitize tumor cuproptosis.^[^ ^34^ ^]^ Another essential trace element, calcium ions, plays an irreplaceable role in maintaining physiological stability, which contributes to electrical signal transmission and nerve conduction.^[^ ^35^ ^]^ Nonetheless, excessive Ca^2+^ accumulation can lead to mitochondrial dysfunction, such as attenuated oxidative phosphorylation and adenosine triphosphate (ATP) production,^[^ ^36^, ^37^ ^]^ which cut off the energy supply of efflux channels, thereby increasing intracellular copper ion levels.

**Scheme 1:**
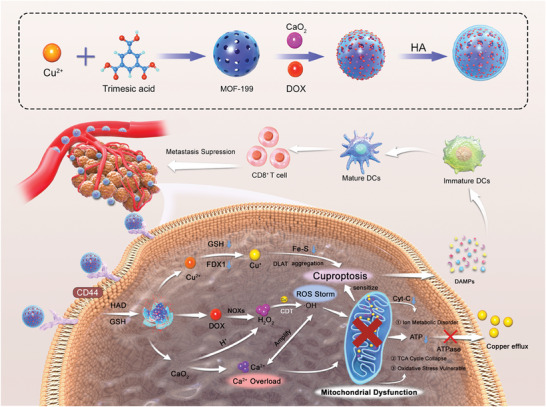
A) Synthesis of the HA‐CD@MOF NPs. B) Schematic illustration of a self‐reinforced bimetallic Mito‐Jammer for precise antitumor therapy based on ROS‐enhanced Ca^2+^ overload‐promoted cascade mitochondrial dysfunction and cuproptosis.

Herein, we propose a Mito‐Jammer to induce a cascade of mitochondrial damage by bimetallic ions to simultaneously boost cuproptosis and immunogenic death (**Scheme** **1**B). To construct the Mito‐Jammer (also known as HA‐CD@MOF), a one‐pot method was employed to integrate the metal‐organic framework (MOF‐199), doxorubicin (DOX), and calcium peroxide (CaO_2_). With the modification of hyaluronate acid (HA) on its surface, the Mito‐Jammer can precisely target mitochondrial aerobic respiration‐dependent persistent tumor cells through specific recognition of the CD44 protein.^[^ ^38^, ^39^ ^]^ Subsequently, the collapse of the Mito‐Jammer in the tumor microenvironment (TME), where glutathione (GSH) and hyaluronidase (HAD) are overexpressed, could release CaO_2_, DOX, and Cu^2+^,^[^ ^40^, ^41^ ^]^ and the exposed CaO_2_ and DOX could further yield hydrogen peroxide (H_2_O_2_) and Ca^2+^ in a weakly acidic environment, strengthening the Cu^2+^‐based Fenton‐like reaction.^[^ ^42^ ^]^ Moreover, the occurrence of Ca^2+^ overload‐exacerbated ROS storms and mitochondrial damage results in the downregulation of intracellular ATP levels and the blocking of Cu‐ATPase. Combined bimetallic ion therapy significantly enhanced protein lipoylation and copper content to facilitate the cuproptosis of tumor cells. Additionally, the Mito‐Jammer promotes the release of damage‐associated molecular patterns (DAMPs), initiating immunogenic cell death (ICD) and effectively suppressing tumor metastasis.^[^ ^43^, ^44^, ^45^ ^]^ Finally, Cu^2+^ could mediate biological tissue ultrasonic conversion and transmit radiofrequency signals to further realize photoacoustic imaging (PAI) and T1‐weighted magnetic resonance imaging (MRI), which perfectly meet the needs of the “all in one” strategy to realize tumor theranostics.^[^ ^46^, ^47^ ^]^ Our work presents a model for the revolution of ion‐triggered cell death through a cascade of mitochondrial damage based on Ca^2+^ overload‐sensitized cuproptosis, holding significant potential for advancing the field of targeted cancer therapies.

## 2 Results and Discussion

### 2.1 Preparation and Characterization of Mito‐Jammer

**Figure 1:**
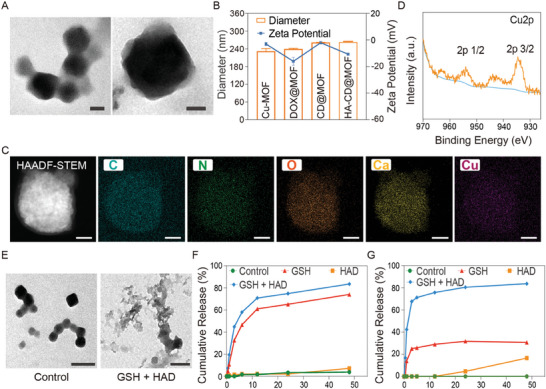
Synthesis and characterization of HA‐CD@MOF NPs. A) TEM images of HA‐CD@MOF NPs (scale bars: 50 nm). B) Diameters and zeta potentials of Cu‐MOF NPs, DOX@MOF NPs, CD@MOF NPs, and HA‐CD@MOF NPs; *n* = 3 . C) High‐angle annular dark field‐scanning transmission electron microscopy (HAADF‐STEM) image and elemental mapping of HA‐CD@MOF NPs (scale bars: 100 nm). D) Cu 2p X‐ray photoelectron spectroscopy (XPS) spectrum of HA‐CD@MOF NPs. E) TEM images of HA‐CD@MOF NPs with different treatments after 24 h (scale bars: 200 nm). F) Ca^2+^ release and G) DOX release from HA‐CD@MOF NPs under different conditions. Results are presented as means ± SD. *p < 0.05, **p < 0.01, ***p < 0.001, ****p < 0.0001.

To prepare the bimetallic Mito‐Jammer, HA‐CD@MOF nanoparticles (NPs) were synthesized using a simple one‐pot method, as illustrated in Scheme 1A. Transmission electron microscopy (TEM) images revealed that HA‐CD@MOF exhibited a regular morphology with uniform size (**Figure** **1**A). As evidenced by the dynamic light scattering examination, the average particle size of HA‐CD@MOF was 263 ± 2.138 nm (PDI: 0.097), and the zeta potential results demonstrated that the surface charge of the NPs varied according to the successful loading of DOX, CaO_2_, and HA (Figure 1B and Figure S2, Supporting Information). Modification of HA onto the surface of Mito‐Jammers was confirmed, and the relative amount of HA was calculated as 5.39% after thermogravimetric analysis, as shown in Figure S3, Supporting Information. Additionally, the color changes of the different NP aqueous solutions confirmed the encapsulation of DOX and CaO_2_ (Figure S4, Supporting Information). The stability of HA‐CD@MOF was investigated in various media including water, phosphate‐buffered saline, fetal bovine serum, and RPMI 1640. The size distributions in all of the above media were well maintained within 13 days of incubation, indicating the exceptional stability of HA‐CD@MOF (Figure S5, Supporting Information). Furthermore, the corresponding elements in HA‐CD@MOF, such as nitrogen, oxygen, calcium, and copper, were all present in the elemental mapping images (Figure 1C). Similarly, the results of X‐ray photoelectron spectroscopy also demonstrated the existence of Ca^2+^ and Cu^2+^ in HA‐CD@MOF (Figure 1D and Figure S6, Supporting Information). Finally, the entrapment efficiencies of DOX and CaO_2_ within HA‐CD@MOF were determined via UV–vis and inductively coupled plasma–mass spectrometry (ICP–MS) to be ≈88.18% and 86.38%, respectively. Altogether, by showing the efficient loading of DOX and CaO_2_, as well as the successful HA modification, the aforementioned results not only demonstrated the successful assembly of the bimetallic Mito‐Jammer but also laid a foundation for its potential for subsequent TME response.

Research has shown that HAD overexpressed in tumor cells can hydrolyze HA, and that high GSH levels spontaneously interact with Cu‐MOF.^[^ ^48^, ^49^ ^]^ Therefore, we speculated that Mito‐Jammer could achieve autogenic degradation in the TME. Consequently, we first investigated the morphological changes in HA‐CD@MOF dispersed in simulated TME solutions at different time points. TEM images showed that the basic morphology of HA‐CD@MOF disappeared when immersed in the GSH/HAD (10 mm, 100 U mL^−1^) solution for 24 h, indicating that the simulated TME triggered the decomposition of the Mito‐Jammer structure (Figure 1E). Subsequently, the tumor‐specific release profiles of the chemotherapy drugs and bimetallic ions were examined in vitro. Within 48 h, the cumulative release rates of DOX, Ca^2+^, and Cu^2+^ reached 83.66%, 82.78%, and 26.68%, respectively, under GSH/HAD stimulation, which were significantly higher than those in the other groups (Figure 1F,G and Figure S7, Supporting Information). Similarly, the gradual orange color change of the solution also confirmed that the dual responses accelerated drug release, consistent with the above results (Figure S8, Supporting Information). The drug release results also revealed that HAD has a limited effect on the decomposition of MOF‐199. Only in TME where GSH and HAD both overexpressed the HA‐CD@MOF could be degraded, thus avoiding the pre‐release of drugs. Therefore, we hypothesized that TME‐triggered Mito‐Jammer decomposition exacerbates the in situ aggregation of bimetallic ions for cascade mitochondrial damage to sensitize tumor cells to cuproptosis.

### 2.2 Simulated TME‐Enhanced CDT and Targeting Abilities of the Mito‐Jammer

**Figure 2:**
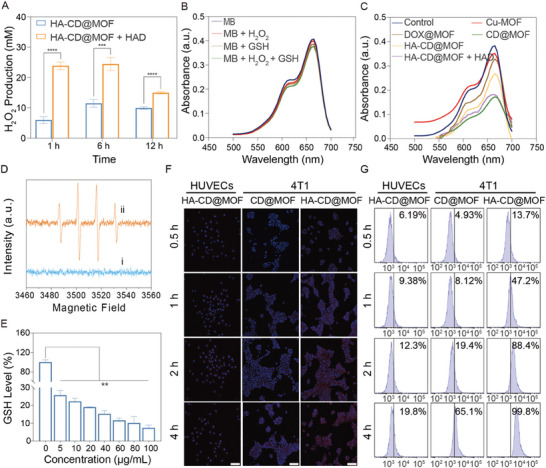
CDT capacity and targeting abilities of HA‐CD@MOF NPs. A) H_2_O_2_‐generating ability of the HA‐CD@MOF NPs with or without HAD in solutions with GSH (10 mm); *n* = 3. B) The effect of GSH (10 mm) and H_2_O_2_ (10 mm) on MB degradation. C) MB degradation rate under different conditions in solutions with GSH (10 mm) and H_2_O_2_ (10 mm). D) Electron spin resonance spectra of DMPO mixed with HA‐CD@MOF NPs under different conditions (i, without GSH and HAD; ii, with GSH and HAD). E) The GSH‐depleting ability of HA‐CD@MOF NPs at different concentrations, *n* = 3. F) CLSM images of HUVECs and 4T1 cells after coincubation with CD@MOF NPs or HA‐CD@MOF NPs for various times (scale bars: 50 µm). G) FCM analysis of the intracellular uptake of CD@MOF NPs or HA‐CD@MOF NPs in 4T1 cells and HUVECs for 0.5, 1, 2, and 4 h. Results are presented as means ± SD. *p < 0.05, **p < 0.01, ***p < 0.001, ****p < 0.0001.

Recent studies revealed that the Cu^2+^‐based Fenton‐like reaction initiates mitochondrial Ca^2+^ buffer dysfunction, indicating that establishing a robust CDT system for the Mito‐Jammer is crucial for implementing tumor bimetallic ion interference. However, the limited levels of H_2_O_2_ within tumor cells limit Fenton‐like reactions. CaO_2_, which is known for its fantastic H_2_O_2_ supply ability, can react with H_2_O to produce H_2_O_2_, particularly under acidic conditions. Therefore, we evaluated the self‐supplied H_2_O_2_ performance of the Mito‐Jammer in a slightly acidic environment. The H_2_O_2_ concentration in HA‐CD@MOF + GSH solutions was 5.95 and 11.45 mm within 1 and 6 h, respectively. In contrast, the H_2_O_2_ production rate increased by 4.01 and 2.13 times after 1 and 6 h of additional HAD treatment, respectively (**Figure** **2**A). This phenomenon implies that the Mito‐Jammer realizes a specific self‐supply of H_2_O_2_ in tumor cells to promote Cu^2+^‐based Fenton‐like reactions. Encouraged by the preeminent H_2_O_2_ production ability, we next assessed the ·OH‐generating activity of the Mito‐Jammer via methylene blue (MB). As shown in Figure 2B,C, GSH, H_2_O_2_, or Cu‐MOF alone exhibited minimal effects on MB degradation, and CD‐MOF led to a remarkable ·OH‐generating effect, which was attributed to the self‐supplied H_2_O_2_ capacity of CaO_2_. However, because of the presence of the HA shell, the degree of MB degradation was suppressed in HA‐CD@MOF without HAD, whereas the HA‐CD@MOF + HAD group presented a downward trend similar to that of the CD‐MOF group, indicating that HA modification can alleviate drug leakage from HA‐CD@MOF. In addition, the coexistence of H_2_O_2_ and CaO_2_ led to the most significant MB degradation via a Fenton‐like reaction mediated by Cu^2+^ (Figure S9, Supporting Information). Apart from the MB experiment, electron spin resonance spectroscopy was also employed to ascertain the ·OH generation of the Mito‐Jammer. The activated Mito‐Jammer (GSH + HAD) showed more intense ·OH signals than the untreated group (Figure 2D). Altogether, it can be summarized that the Mito‐Jammer specifically exerts its CDT function under GSH and HAD activation. Since GSH is involved in Fenton‐like reactions and serves as the primary intracellular reductive species, its depletion might reflect CDT and boost therapeutic efficacy. Accordingly, the GSH‐depletion ability of HA‐CD@MOF was determined via a 5,5′‐dithiobis‐(2‐nitrobenzoic acid) (DTNB) indicator. The GSH content decreased as the Mito‐Jammer concentration increased (Figure 2E), indicating that GSH can be depleted. These data corroborate that the Mito‐Jammer can activate CDT in tumor cells in situ in a self‐reinforced manner.

Highly enriched NPs within the tumor cells are a prerequisite for CDT. HA can bind specifically to CD44 receptors overexpressed on the surface of tumors, endowing the Mito‐Jammer with effective tumor endocytosis. Thus, the cellular uptake of Mito‐Jammer was assessed using confocal laser scanning microscopy (CLSM) and flow cytometry (FCM). Briefly, the red fluorescence from DOX in HA‐CD@MOF was amplified with the extension of culture time in 4T1 cells, while the fluorescence intensity of CD@MOF was significantly weakened. Moreover, faint red fluorescence was observed in human umbilical vein endothelial cells (HUVECs) treated with HA‐CD@MOF for 4 h compared to 4T1 cells (Figure 2F), indicating that HA promoted the endocytosis of the Mito‐Jammer in tumor cells. In addition, similar cellular uptake performance was verified via FCM (Figure 2G). Furthermore, the lysosomal compartments were stained with a LysoTracker probe to show the intracellular distribution of free DOX and HA‐CD@MOF in 4T1 cells. The red fluorescence emitted from free DOX outlined the shape of the nuclei but barely overlapped with the lysosomes (green fluorescence), whereas a large portion of the red fluorescence was co‐localized in the lysosomes in the HA‐CD@MOF group, signifying that the Mito‐Jammer was endocytosed and distributed in the lysosomes (Figure S10, Supporting Information). In addition, biological transmission electron microscopy (bio‐TEM) was used to observe the uptake and degradation of HA‐CD@MOF by 4T1 cells. As shown in Figure S11, Supporting Information, HA‐CD@MOF with a regular morphology was observed in the 4T1 cells, indicating that HA‐CD@MOF was endocytosed by the 4T1 cells after 1 h incubation. In contrast, at 2 h, HA‐CD@MOF was observed in the cytoplasm or incomplete vesicles with ambiguous boundaries, and the morphology of the NPs became incompact. In addition, HA‐CD@MOF was fully degraded by 4T1 cells at 12 h. Thus, we hypothesized that the presence of HAD and GSH within the tumor cells triggered the decomposition of the Mito‐Jammer. Collectively, these findings certify that the Mito‐Jammer can efficiently aggregate within tumor cells to exert TME‐enhanced CDT, laying a foundation for the subsequent cascade of mitochondrial damage to sensitize cuproptosis.

### 2.3 Self‐Reinforced CDT Using Mito‐Jammer in the TME

**Figure 3:**
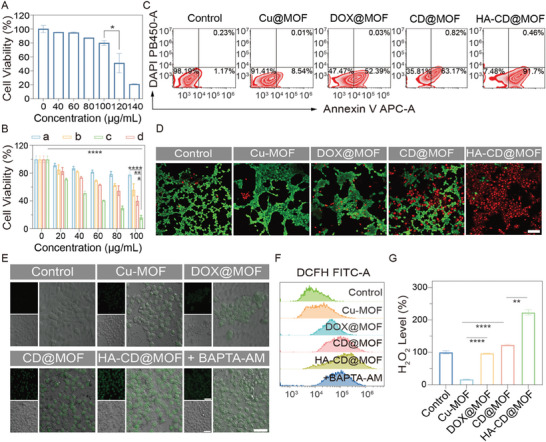
Self‐reinforced chemodynamic potency of the Mito‐Jammer in the TME. A) Biosafety of HUVECs after coincubation with different concentrations of HA‐CD@MOF NPs for 12 h, *n* = 3. B) Cell viability of 4T1 cells after treatment with a) Cu‐MOF, b) DOX@MOF, c) CD@MOF, and d) HA‐CD@MOF NPs at different concentrations for 6 h, *n* = 3. C) FCM measurement of 4T1 cell apoptosis ratios in the control, Cu‐MOF, DOX@MOF, CD@MOF, and HA‐CD@MOF NP groups. D) CLSM images of 4T1 cells after live and dead staining. Red fluorescence refers to dead cells, and green fluorescence refers to live cells (scale bar: 50 µm). E) CLSM images of DCFH‐DA assays of 4T1 cells under different treatments (scale bars: 50 µm). F) Quantitative analysis of DCF fluorescence intensity within 4T1 cells measured via FCM. G) H_2_O_2_ levels within 4T1 cells under various conditions, *n* = 3. Results are presented as means ± SD. *p < 0.05, **p < 0.01, ***p < 0.001, ****p < 0.0001.

Encouraged by the simulated TME‐enhanced ·OH generation efficiency of the Mito‐Jammer, we next explored its antitumor effects at the cellular level. First, the biocompatibility and cytotoxicity of the Mito‐Jammer were measured using the CCK‐8 assay. HUVECs were incubated with HA‐CD@MOF at various concentrations for 12 h; as a result, even when the concentration of the Mito‐Jammer increased to 100 µg mL^−1^, it still presented negligible toxicity toward HUVECs (**Figure** **3**A), showing good biosafety. The viability of 4T1 cells sharply decreased (from 71.36% to 16.44%) as the concentration of NPs increased, which was ascribed to the high tumor affinity triggered by the TME‐enhanced toxicity. Furthermore, to objectively analyze the role of the different components in Mito‐Jammer, we synthesized Cu‐MOF, DOX@MOF, and CD@MOF to investigate the biological functions of DOX, CaO_2_, and HA. The absence of any constituents resulted in an attenuated antitumor effect (Figure 3B), validating the reasonable assembly of Mito‐Jammer to collaboratively inhibit tumor growth. Apoptosis was further detected via FCM. At the same concentration, the highest apoptotic rate of 4T1 cells was observed in the HA‐CD@MOF group (up to 91.71%), significantly increasing to 10.76‐fold, 1.75‐fold, and 1.45‐fold higher than that in the Cu‐MOF, DOX@MOF, and CD@MOF groups, respectively (Figure 3C). To visually estimate the therapeutic efficacy of the NPs, we performed calcein‐AM and propidium iodide staining of 4T1 cells to differentiate between live and dead cells. The images captured from this staining showed that Mito‐Jammer improved the number of red spots, whereas the other groups mainly consisted of surviving green cells (Figure 3D). Overall, these findings illustrate that the Mito‐Jammer fully exerted its inherent TME characteristics to amplify the superiority of bimetallic ions for tumor elimination.

Our in vitro experiments have revealed that CaO_2_ is a great agent to enhance the Fenton‐like reaction. DOX could also activate nicotinamide adenine dinucleotide phosphate oxidases (NOXs) to convert oxygen (O_2_) into ·O_2_^−^ and endogenous H_2_O_2_ via superoxide dismutase employing disproportionation reactions, thus strengthening the CDT capacity of Mito‐Jammer.^[^ ^50^, ^51^ ^]^ To determine the detailed mechanism of the TME‐oriented self‐reinforced bimetallic antitumor effect, we investigated intracellular ROS levels using the green fluorescence of the 2,7‐dichlorodihydrofluorescein diacetate (DCFH‐DA) probe. As shown in Figure 3E, there was no visible green fluorescence in the control group treated with 1640 culture medium and the Cu‐MOF group, while enhanced green fluorescence could be observed in the DOX@MOF and CD@MOF groups, suggesting that the H_2_O_2_ provided by DOX and CaO_2_ markedly promoted ·OH generation. As expected, the 4T1 cells treated with HA‐CD@MOF exhibited brilliant green fluorescence. As Ca^2+^ overload‐mediated mitochondrial damage can upregulate intracellular ROS levels, 4T1 cells were treated with BATPA‐AM, a Ca^2+^ chelator, and Mito‐Jammer to assess ROS production. Compared to the Mito‐Jammer group, the introduction of BATPA‐AM downregulated green fluorescence expression, suggesting that CDT and Ca^2+^ overload triggered by bimetallic ions jointly led to ROS explosion. Furthermore, the FCM results for DCFH‐DA matched well with the CLSM images (Figure 3F and Figure S12, Supporting Information). Next, to show that intracellular H_2_O_2_ levels are closely related to ROS synthesis, the concentration of H_2_O_2_ in 4T1 cells treated with various compounds was determined. As shown in Figure 3G, the Cu‐MOF group dramatically reduced the intracellular H_2_O_2_ content, because the Fenton‐like reaction requires the consumption of H_2_O_2_. However, benefitting from the robust self‐supplying H_2_O_2_ capacity of DOX and CaO_2_, the H_2_O_2_ level in 4T1 cells treated with DOX@MOF, CD@MOF, and HA‐CD@MOF was elevated, exhibiting the self‐reinforced CDT potency of the Mito‐Jammer. Accordingly, the above data indicate that the Mito‐Jammer can dynamically regulate TME components to realize self‐reinforced CDT.

### 2.4 Mito‐Jammer‐Mediated Cascade Mitochondrial Damage to Sensitize Cuproptosis

**Figure 4:**
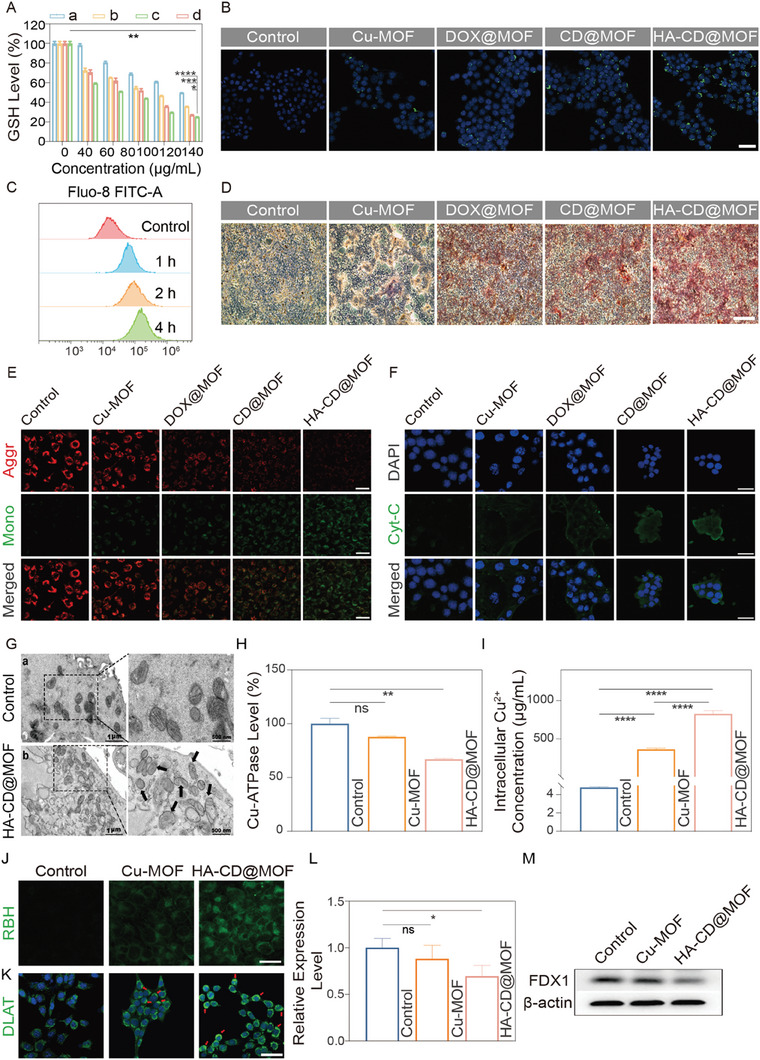
Evaluation of cascade mitochondrial injury sensitization to cuproptosis caused by HA‐CD@MOF NPs. A) GSH levels in 4T1 cells after different treatments: a) Cu‐MOF, b) DOX@MOF, c) CD@MOF, d) HA‐CD@MOF, *n* = 3. B) CLSM images of intracellular uptake of Ca^2+^ in 4T1 cells after different treatments (scale bar: 50 µm). C) FCM analysis of Ca^2+^ levels in 4T1 cells after incubation with HA‐CD@MOF NPs at various time points. D) Identification of exocytosis products of tumor cells via Alizarin Red staining (scale bar: 500 µm). E) CLSM images of JC‐1‐stained 4T1 cells under different conditions (scale bars: 50 µm). F) CLSM images of Cyt‐C in 4T1 cells treated with control, Cu‐MOF, DOX@MOF, CD@MOF, and HA‐CD@MOF NPs (scale bars: 50 µm). G) Bio‐TEM of mitochondria in a) untreated 4T1 cells and b) HA‐CD@MOF NP‐treated 4T1 cells. H) Cu‐ATPase activity after different treatments; *n* = 3. I) Intracellular Cu^2+^ concentration after different treatments; *n* = 3. J) CLSM images of intracellular Cu^2+^ accumulation after treatment with the control, Cu‐MOF, and HA‐CD@MOF NPs in 4T1 cells (scale bars: 25 µm). K) CLSM images of DLAT aggregation (red arrows) in 4T1 cells after treatment with control, Cu‐MOF, and HA‐CD@MOF NPs (scale bar: 50 µm). L) The corresponding quantitative analysis of FDX1 protein expression based on western blotting results, *n* = 3. M) Western blotting results of cuproptosis markers in 4T1 cells treated with control, Cu‐MOF, and HA‐CD@MOF NPs. Results are presented as means ± SD. *p < 0.05, **p < 0.01, ***p < 0.001, ****p < 0.0001.

Recent studies have shown that cuproptosis is a copper‐dependent cell death driven by mitochondrial damage and subsequent mitochondrial protein stress.^[^ ^52^ ^]^ However, high expression of GSH and the presence of the copper transporter family in tumor cells impede the occurrence of cuproptosis.^[^ ^53^, ^54^ ^]^ Considering that GSH can be converted to oxidized glutathione in a Fenton‐like reaction, the GSH depletion capability of the Mito‐Jammer may contribute to cuproptosis.^[^ ^55^ ^]^ Thus, intracellular GSH levels were examined using the CheKine Reduced Glutathione Colorimetric Assay Kit. As shown in **Figure** **4**A, all NPs exhibited concentration‐dependent GSH depletion in 4T1 cells. Notably, HA‐CD@MOF presented the most significant reduction in GSH levels among these groups, thus overcoming the first obstacle to cuproptosis. In addition, the Cu‐ATPase transfers copper ions to the extracellular space to prevent their accumulation through ATPase hydrolysis.^[^ ^56^, ^57^ ^]^ Mitochondria are the main organelles of ATP production, and damage to these organelles can reduce the efflux ability of Cu‐ATPase. Although TME‐reinforced CDT elevated ·OH generation,mitochondrial destruction was still limited. A persistent increase in intracellular Ca^2+^ (identified as Ca^2+^ overload) could reportedly trigger the opening of cyclophilin D‐dependent mitochondrial membrane pores,^[^ ^58^ ^]^ indicating impaired mitochondrial biological function. Hence, we estimated intracellular Ca^2+^ concentration using a Ca^2+^ probe (Fluo‐8 AM). The CLSM image demonstrated that green fluorescence (representing free Ca^2+^) within the 4T1 cells treated with CD@MOF and HA‐CD@MOF was the strongest, which was evidently higher than that of the other groups (Figure 4B). Interestingly, this trend aligned with the DCFH‐DA results, implying that Ca^2+^ accumulation facilitated ROS production. Additionally, FCM analysis revealed a positive correlation between intracellular Ca^2+^ levels and the incubation time of the NPs (Figure 4C and Figure S13, Supporting Information). To demonstrate the performance of Mito‐Jammer in boosting Ca^2+^ overload, Alizarin Red staining was performed to observe cellular calcification. The red calcification area in 4T1 cells increased in a time‐dependent manner (Figure 4D), further confirming that the mitochondrial damage mechanism depends on Ca^2+^ metabolic dysfunction and CDT.

Based on the above findings, we assessed the extent of mitochondrial damage in 4T1 cells using the 6‐tetrachloro‐1,1,3,3‐tetraethylbenzimidazolylcarbocyanine iodide (JC‐1) probe. The fluorescence of JC‐1 transitioning from red to green represents decreased mitochondrial membrane potential (MMP). Apparent red fluorescence was observed in the control and Cu‐MOF‐treated groups. In contrast, the cells treated with HA‐CD@MOF emitted bright green and faint red fluorescence (Figure 4E). Similarly, treatment with the Mito‐Jammer resulted in a considerable loss of cellular MMP, as evidenced by the 61.58% negative potential in FCM (Figure S14, Supporting Information), signifying that bimetallic therapy resulted in remarkable mitochondrial damage. Cytochrome C (Cyt‐C) is a protein embedded in the inner membrane of the mitochondria, and its extravasation is a hallmark of early mitochondrial damage. Thus, we used immunofluorescence staining to monitor the release of Cyt‐C from the mitochondria. As shown in Figure 4F, CD@MOF and HA‐CD@MOF treatments induced strong green fluorescence (representing Cyt‐C) in these groups, suggesting that CD@MOF coordinated with Ca^2+^ overload‐initiated mitochondrial apoptosis. To further visualize the morphological changes in the mitochondria, 4T1 cells subjected to Mito‐Jammer were inspected using bio‐TEM. Notably, a swollen morphology and an expanded cavity were observed in the mitochondria of 4T1 cells treated with Mito‐Jammer, which were clearly distinct from those observed in the control sample (Figure 4G). After verifying the mitochondrial damage, we evaluated its biological function in ATP production. The intracellular ATP concentration was inhibited in the Mito‐Jammer group, and the relative ATP level was reduced to 49% of that in the control group (Figure S15, Supporting Information), indicating the possibility of cutting off the energy source of Cu‐ATPase to weaken copper efflux. Accordingly, the Cu‐ATPase activity of the tumor cells was investigated using a Cu‐ATPase assay kit. As shown in Figure 4H, the ATPase activity on the cell membrane was slightly reduced after incubation with Cu‐MOF. However, the Mito‐Jammer treatment caused a substantial decline in activity to 66.95% of that of the control group. The blockade of the copper transporter channel inevitably led to intracellular copper ion metabolic disorder, and the intracellular Cu^2+^ concentration after various treatments was verified. The ICP‒MS results showed that compared with the Cu‐MOF group (362.91 ng mL^−1^), 4T1 cells treated with Mito‐Jammer exhibited a much higher increase in Cu^2+^ to 816.90 ng mL^−1^, which was 170.54 times that of the control group (4.79 ng mL^−1^, Figure 4I). In addition, a Cu^2+^ probe was used to determine changes in intracellular copper levels. (Figure 4J). As expected, the Mito‐Jammer group strongly initiated Cu^2+^ accumulation, consistent with the ICP‐MS results, demonstrating that the Mito‐Jammer could block the copper efflux by cutting the energy supply of ATPase via mitochondrial dysfunction. These data reveal that the dual obstacles of GSH enrichment and copper ion homeostasis were settled by the TME‐activatable Mito‐Jammer for sensitizing cuproptosis.

Dihydrolipoamide S‐acetyltransferase (DLAT) and ferredoxin 1 (FDX1) are the principal cuproptosis‐related genes. The former combines with copper ions to form oligomers, while the latter is an upstream regulator of protein lipoylation in the mitochondria; together, they can cause proteotoxic stress, ultimately inducing cuproptosis. First, we studied DLAT oligomerization using CLMS (Figure 4K). DLAT aggregation in cells treated with Cu‐MOF has already been observed, whereas cells treated with Mito‐Jammer exhibited a much higher degree of DLAT foci. Furthermore, western blotting was performed to evaluate changes in FDX1. The gray value of the FDX1 protein decreased by ≈11.86% and 30.46% with the Cu‐MOF and HA‐CD@MOF treatments, respectively (Figure 4L,M). These results emphasize the superiority and practicality of Mito‐Jammer in enhancing cuproptosis in a Cu^2+^/Ca^2+^ collaborative manner.

### 2.5 Targeting, Biodistribution, and Bioimaging of Mito‐Jammer In Vivo

**Figure 5:**
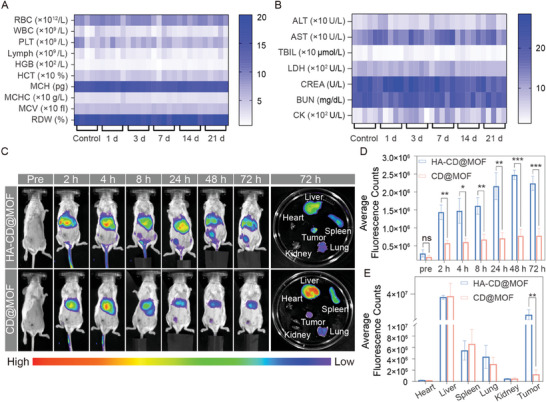
Biosafety and tumor‐targeting assay of HA‐CD@MOF in vivo. A) Routine blood (left) and B) blood biochemistry (right) analysis of mice sacrificed on certain days after HA‐CD@MOF NP treatment; *n* = 5. C) Fluorescence images of mice treated with CD@MOF NPs and HA‐CD@MOF NPs at different time points in vivo and ex vivo fluorescence images of the main organs and tumors harvested from mice at 72 h. D) Quantitative analysis of fluorescence intensity at the tumor site in vivo; *n* = 3. E) Quantitative analysis of fluorescence intensity in the main organs and harvested tumors ex vivo; *n* = 3. The results are presented as means ± SD. *p < 0.05, **p < 0.01, ***p < 0.001, ****p < 0.0001.

Inspired by the excellent tumor cell targeting, outstanding mitochondrial damage, and significant cuproptosis‐sensitizing capabilities of the Mito‐Jammer, we further tested its performance in vivo. Prior to in vivo experiments, the biocompatibility and biosafety of HA‐CD@MOF were evaluated. Healthy BALB/c mice were intravenously injected with Mito‐Jammer on days 1, 3, 7, 14, and 28, while the control group was administered saline. Blood and major organs of the mice were collected for analysis. Biochemical and routine blood test analyses indicated no evident differences between mice at various time points (**Figure** **5**A,B). Additionally, hematoxylin‐eosin (H&E) staining of the major organs showed almost no histopathological changes (Figure S16, Supporting Information), demonstrating the high biosafety of the Mito‐Jammer for subsequent in vivo treatment.

To study the biodistribution and tumor accumulation of Mito‐Jammer in live mice in real time, fluorescence imaging (FLI) analysis was conducted. Notably, HA prolonged the retention time and boosted the accumulation of HA‐CD@MOF in tumor tissues, and a fluorescence peak was observed at 48 h (Figure 5C,D). In ex vivo bioimaging, HA‐CD@MOF showed a ≈3.05‐fold increase in the fluorescence signal within the tumor compared to that of CD@MOF, and the tumor region became the main enrichment site in the body after the liver (Figure 5E). Based on these results, we concluded that the Mito‐Jammer possesses superior tumor accumulation efficiency owing to the specific binding of CD44 overexpressed on the tumor cell membrane with HA.

**Figure 6:**
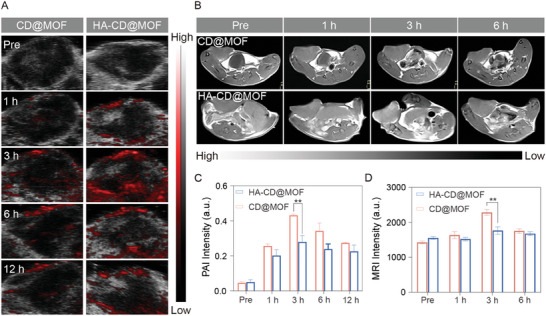
PAI/MRI ability of HA‐CD@MOF NPs. A) PAI images of the tumor after treatment with CD@MOF NPs and HA‐CD@MOF NPs. B) T_1_‐weighted images of the tumor at various time points after intravenous injection of CD@MOF NPs and HA‐CD@MOF NPs. C) Quantitative analysis of PAI intensity of the tumor; *n* = 3. D) T1‐weighted MRI signal intensity of the tumor; *n* = 3. Results are presented as means ± SD. *p < 0.05, **p < 0.01, ***p < 0.001, ****p < 0.0001.

Considering the unique degradation of the Mito‐Jammer in the GSH/HAD‐overexpressing TME to release Cu^2+^, the TME‐activatable imaging potential in both PAI and T_1_‐weighted MRI was investigated. First, the concentration‐dependent linear relationship of the Mito‐Jammer with both the PAI value (R^2^ = 0.9989, Figure S17, Supporting Information) and the r_1_ value in vitro (R^2^ = 0.9911, Figure S18, Supporting Information) was calculated. HA‐CD@MOF and CD@MOF were intravenously injected into 4T1 tumor‐bearing BALB/c mice to study the imaging signal discrepancy within the tumor tissue. The captured photoacoustic images and statistical analysis revealed that HA‐CD@MOF presented the strongest photoacoustic signal in tumor tissue at 3 h, and HA equipped it with better tumor‐targeting potency than the CD@MOF group at each time point (**Figure** **6**A,C). Furthermore, the r_1_ value of Cu^2+^ released from HA‐CD@MOF upon GSH and HAD treatment was 1.269 s^−1^·mM^−1^, confirming its potential as a TME‐activatable T_1_‐weighted MRI agent (Figure S18, Supporting Information). Moreover, the in vivo images showed an enhanced T_1_‐weighted MRI signal in the HA‐CD@MOF group, which peaked at 3 h (Figure 6B,D). Based on the imaging results, the HA‐modified Mito‐Jammer may provide insight into the tumor monitoring of copper‐based nanoagents with high sensitivity and specificity.

### 2.6 Antitumoral Effects of Mito‐Jammer In Vivo

**Figure 7:**
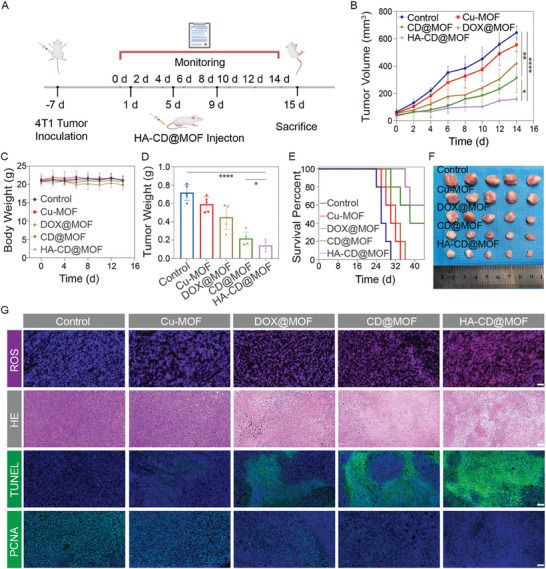
Antitumor effects of HA‐CD@MOF NPs in vivo. A) Therapeutic process of HA‐CD@MOF NPs. B) Average tumor growth curves of tumor‐bearing mice after various treatments; *n* = 5. C) Body weight change curves of tumor‐bearing mice receiving different treatments; *n* = 5. D) Average weights of tumor excised from 4T1‐tumor‐bearing mice at day 15; *n* = 5. E) Survival curves of mice after various treatments; *n* = 5. F) Digital photos of harvested tumors; *n* = 5. G) ROS, H&E, terminal deoxynucleotidyl transferase dUTP nick‐end labeling (TUNEL), and proliferating cell nuclear antigen (PCNA) staining of tumor tissues from mice (scale bars: 100 µm). Results are presented as means ± SD. *p < 0.05, **p < 0.01, ***p < 0.001, ****p < 0.0001.

The available in vitro therapeutic accomplishment and biocompatibility of Mito‐Jammer encouraged us to investigate its antitumor performance in vivo in 4T1 tumor‐bearing mouse models (**Figure** **7**A), which were randomly divided into five groups (*n* = 5) for various therapies: control, Cu‐MOF, DOX@MOF, CD@MOF, and HA‐CD@MOF. As shown in Figure 7B, a slight discrepancy in tumor volume was observed between the control and Cu‐MOF groups, indicating that introducing excessive Cu^2+^ was not sufficiently effective in inhibiting tumor growth. However, benefitting from the enhanced Fenton‐like reaction and bimetallic ion therapy, the DOX@MOF and CD@MOF groups showed an enhanced advantage in tumor growth inhibition (TGI) over Cu‐MOF treatment. In addition, the HA‐CD@MOF group exhibited considerable therapeutic effect in tumor suppression. After sacrifice at the end of the experiment, the excised tumors were collected for weighing and photography. The tumor weight in the control group was ≈0.72 g, which was 3.42 and 5.14 times higher than those in the CD@MOF and HA‐CD@MOF groups, respectively (Figure 7D). The ex vivo tumor photographs confirmed these therapeutic effects (Figure 7F). In addition, TGI rates were calculated based on tumor volumes to quantitatively confirm the antineoplastic performance of Mito‐Jammer. The HA‐CD@MOF group exhibited the highest TGI rate (72.45%) after a 14‐day treatment, outperforming the CD@MOF (62.27%), DOX@MOF (49.48%), and Cu‐MOF (26.05%) groups Similarly, the TGI rates based on tumor weight showed a corresponding trend (Figure S19, Supporting Information). We also noted that all groups showed minimal variation in mouse weight during treatment (Figure 7C). Moreover, we carried out a 44‐day monitoring to investigate the effect of the NPs on prolonging the survival time of the mice. The animal survival curves showed that in the HA‐CD@MOF group, 60% of the mice were still alive after 44 days. In contrast, all the mice in the control group died on day 30 and only 20% of the mice in the CD@MOF lived to 44 days (Figure 7E). Furthermore, as shown in Figure 7G, immunofluorescence and H&E staining of the tumor tissues revealed that mice treated with Mito‐Jammer showed the highest accumulation of ROS (red) and the most serious nuclear damage. Terminal deoxynucleotidyl transferase dUTP nick‐end labeling and proliferating cell nuclear antigen immunofluorescence staining suggested that HA‐CD@MOF induced the highest apoptotic rate (green spot) and the lowest proliferative rate. Moreover, H&E staining of the main organs indicated that no abnormalities were observed in any group (Figure S20, Supporting Information), validating that all treatment strategies were biocompatible and well tolerated. Consequently, in vivo treatment strongly corroborates that TME‐activatable CDT and cascade mitochondrial damage‐mediated cuproptosis using Mito‐Jammer inhibits tumor growth.

### 2.7 Immunotherapy and Metastasis Suppression Performance via Mito‐Jammer‐Mediated Cuproptosis

**Figure 8:**
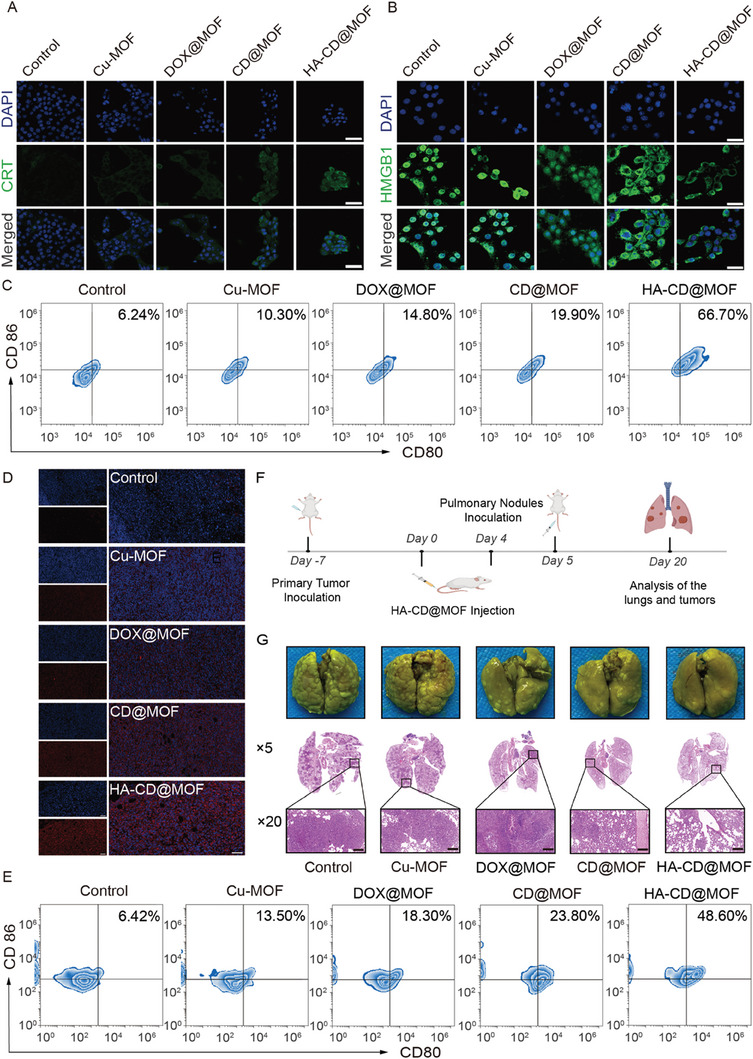
Immunotherapy and metastasis suppression performance of HA‐CD@MOF NPs. CLMS images of A) CRT expression and B) HMGB1 release profiles of 4T1 cells (scale bars: 50 µm). C) FCM analysis of DC maturation stimulated by HA‐CD@MOF NPs in vitro. D) Immunofluorescence staining of CRT expressed in tumor tissues from mice (scale bars: 100 µm). E) DC maturation in vivo measured via FCM. F) Schematic illustration of the lung metastasis suppression experimental process. G) Bouin's tri‐chrome fixed lung tissue of mice after sacrifice and the corresponding H&E staining images (scale bars: 200 µm).

Owing to the outstanding antitumor effects of the Mito‐Jammer and its cuproptosis‐mediated immune arousal capacity,^[^ ^59^, ^60^ ^]^ its immune response was further evaluated in vitro and in vivo. The transfer of calreticulin (CRT) to the cell membrane surface and the secretion of high‐mobility group box protein B1 (HMGB1) outside the tumor cells are hallmarks of ICD. Immunofluorescence staining revealed that Cu‐MOF and DOX@MOF treatments could not form visible CRT on the cell membrane, whereas apparent green fluorescence spots were captured in 4T1 cells treated with CD@MOF and HA‐CD@MOF, suggesting that Ca^2+^ overload‐mediated cuproptosis triggered CRT translocation (**Figure** **8**A). Similarly, a large amount of HMGB1 was released from the 4T1 cells in the HA‐CD@MOF group compared to that in the other treatments (Figure 8B). Moreover, a transwell model was constructed to verify the performance of Mito‐Jammer in inducing dendritic cell (DC) maturation in vitro. FCM results showed enhanced DC maturation in the DOX@MOF (14.80%) and CD@MOF (19.90%) groups, which may be attributed to a cascade‐amplified cuproptosis response. As HA improved NP enrichment in tumor cells, HA‐CD@MOF treatment resulted in the most pronounced DC maturation (66.70%) (Figure 8C and Figure S21, Supporting Information). Subsequently, enzyme‐linked immunosorbent assays (ELISAs) were conducted to assess the secretion levels of pro‐inflammatory factors including IL‐6, TNF‐α, and IFN‐γ. Remarkably, the 4T1 cells co‐incubated with HA‐CD@MOF promoted the secretion of these cytokines to varying degrees (Figure S23, Supporting Information). These data imply that the Mito‐Jammer can evoke immune efficacy by effectively inducing DAMPs release from 4T1 tumor cells and facilitating DC maturation. The corresponding ICD indices were investigated in vivo. Immunofluorescence staining of CRT in tumor slices demonstrated increased positive red fluorescence in the CD@MOF and HA‐CD@MOF groups, indicating the occurrence of cuproptosis‐elicited ICD in vivo (Figure 8D). Tumor FCM analysis revealed a significant increase in the proportion of DCs within the tumors of mice treated with HA‐CD@MOF. Specifically, the detected increase was 7.6‐fold within the tumor compared to that in the control group (6.42%) (Figure 8E and Figure S22, Supporting Information). In addition, the ELISA results revealed that the IL‐6, TNF‐α, and IFN‐γ cytokines in the peripheral blood were the highest in the HA‐CD@MOF treatment (Figure S23, Supporting Information), suggesting that the upregulation of the immune response through the cascade stimulated cuproptosis in vivo.

Furthermore, it has been reported that cuproptosis‐mediated immunotherapy and severe oxidative stress within tumor cells can inhibit the epithelial–mesenchymal transition, an important process in tumor invasion and metastasis, to alleviate the migration and invasion of tumor cells.^[^ ^61^, ^62^, ^63^ ^]^ Satisfied by the immune arousal of Mito‐Jammer‐mediated cuproptosis, the tumor metastasis inhibition performance was further investigated. First, a wound‐healing assay was performed to evaluate the inhibitory effect of Mito‐Jammer on cell invasion migration. The control group exhibited the highest wound healing rate (74.28%), and the Cu‐MOF (63.10%), DOX@MOF (46.48%), and CD@MOF (38.82%) rates declined owing to their enhanced ROS‐generating abilities. Under the synergistic effects of the tumor‐specific ROS storm and the Ca^2+^ overload‐sensitized cuproptosis, the HA‐CD@MOF group had the lowest wound healing rate (12.36%). Second, the transwell assay was conducted to further observe the migration inhibitory effect of Mito‐Jammer on tumor cells. The images and quantitative analysis revealed that HA‐CD@MOF could efficiently suppress tumor cell migration with fewer cells being observed per field, which was 3.25 times fewer than that of the control group (Figure S25, Supporting Information). Then, a mouse model of lung metastasis was established for different treatments, and the lungs were collected to observe the antitumor metastasis effect of the Mito‐Jammer in vivo (Figure 8F). Bouin's staining of the lung tissue visually indicated that the control and Cu‐MOF groups were still filled with metastatic nodules. Consistent with the immune response results, HA‐CD@MOF elicited evident metastatic tumor inhibition with the fewest pulmonary nodules (Figure 8G). Accordingly, H&E‐stained mouse lung sections further verified the antimetastatic capacity of bimetallic ions to sensitize mice to cuproptosis. Moreover, the H&E staining results of the major organs again revealed the biosafety of the Mito‐Jammer by showing no abnormalities in the treated mice of DC maturation and lung metastasis experiments (Figures S25 and S26, Supporting Information). In summary, these findings suggest that the Mito‐Jammer can effectively induce an immune response and establish immune advantages for suppressing tumor metastasis through bimetallic ion cascade mitochondrial dysfunction‐enhanced apoptosis.

## 3 Conclusion

In conclusion, we successfully fabricated a self‐reinforced bimetallic Mito‐Jammer to sensitize cuproptosis and cuproptosis‐related immunotherapy. The presence of an HA shell endowed Mito‐Jammer with excellent tumor‐targeting capabilities and minimal side effects. Upon decomposition within the GSH/HAD‐overexpressing TME, Mito‐Jammer released CaO_2_ and Cu^2+^. The exposed CaO_2_ further yielded H_2_O_2_ and Ca^2+^ in a weakly acidic environment to strengthen the Cu^2+^‐based Fenton‐like reaction. Furthermore, the combination of CDT and Ca^2+^ overload initiated a ROS storm and cascade mitochondrial damage, resulting in the downregulation of intracellular ATP levels and the subsequent blocking of Cu‐ATPase to trigger cuproptosis. Apart from selectively improving the ions aggregation within tumor cells, we described a promising approach for sensitizing tumor cuproptosis using Ca^2+^ overload to block Cu^2+^ efflux by cutting off the energy supply to Cu‐ATPase. Other than studying the ultimate anti‐tumor therapeutic efficacy of the Mito‐Jammer, we explored its potential in immune arousal and metastasis inhibition. Therefore, this study presents a promising approach to bimetallic ion interference for boosting tumor cuproptosis and immunotherapy. Since multiple studies have revealed the correlation between mitochondria dysfunction and tumoral immune response, and cuproptosis is a mitochondria respiration‐dependent cell death pathway, this enhanced cuproptosis strategy could hopefully be investigated in future research based on immunotherapy and mitochondria regulation.

## Conflict of Interest

The authors declare no conflict of interest.

## Supporting information

Supporting Information

## Data Availability

The data that support the findings of this study are available from the corresponding author upon reasonable request.
