# Supplementary material for: Self‐Reinforced Bimetallic Mito‐Jammer for Ca2+ Overload‐Mediated Cascade Mitochondrial Damage for Cancer Cuproptosis Sensitization
Source: Adv Sci (Weinh). 2024 Feb 11;11(15):2306031. doi: 10.1002/advs.202306031 (PMC11022715; doi:10.1002/advs.202306031)
Supplement: Supplementary file 1 — Supporting Information [file ADVS-11-2306031-s001.pdf]

ADVANCED SCIENCE
Open Access

Supporting Information

for *Adv. Sci.*, DOI 10.1002/advs.202306031

Self-Reinforced Bimetallic Mito-Jammer for Ca^{2+} Overload-Mediated Cascade Mitochondrial Damage for Cancer Cuproptosis Sensitization

*Chier Du, Xun Guo, Xiaoling Qiu, Weixi Jiang, Xiaoting Wang, Hongjin An, Jingxue Wang, Yuanli Luo, Qianying Du, Ruoyao Wang, Chen Cheng, Yuan Guo, Hua Teng, Haitao Ran, Zhigang Wang, Pan Li, Zhiyi Zhou and Jianli Ren**

Supporting Information

Self-Reinforced Bimetallic Mito-Jammer for Ca^{2+} Overload-Mediated Cascade Mitochondrial Damage for Cancer Cuproptosis Sensitization

Chier Du, Xun Guo*, Xiaoling Qiu, Weixi Jiang, Xiaoting Wang, Hongjin An, Jingxue Wang, Yuanli Luo, Qianying Du, Ruoyao Wang, Chen Cheng, Yuan Guo, Hua Teng, Haitao Ran, Zhigang Wang, Pan Li, Zhiyi Zhou, Jianli Ren**

C. Du, Dr. X. Guo, Dr. W. Jiang, Dr. X. Wang, H. An, Dr. J. Wang, Dr. Y. Luo, Dr. C. Cheng, Dr. Y. Guo, Dr. H. Teng, Prof. H. Ran, Prof. Z. Wang, Prof. P Li, Prof. J. Ren

Department of Ultrasound and Chongqing Key Laboratory of Ultrasound Molecular Imaging, the Second Affiliated Hospital of Chongqing Medical University, Chongqing 400010, P. R. China.

Email: renjianli@cqmu.edu.cn (J. Ren).

Dr. X. Qiu

Department of Intensive Care Unit, the Second Affiliated Hospital of Chongqing Medical University, Chongqing 400010, P. R. China.

Qianying Du

Department of Radiology, Second Affiliated Hospital of Chongqing Medical University, Chongqing 400010, P. R. China.

Ruoyao Wang

Department of Breast and Thyroid Surgery, Second Affiliated Hospital of Chongqing Medical University, Chongqing 400010, P. R. China.

Zhiyi Zhou

Department of General Practice, Chongqing General Hospital, Chongqing 400010, P. R. China.

[#]These authors contributed equally to this work.

Supplementary methods

Experimental materials and reagents. Trimesic acid (98%), triethylamine (⩾99%), copper nitrate trihydrate (99.9%) and calcium chloride ($CaCl_2$, 96%) were purchased from Shanghai Macklin Biochemical Co., Ltd. Doxorubicin (DOX，97%), hyaluronic acid (HA, Mw=5000-150000), glutathione (GSH, 98%), 5,5′-Dithio bis-(2-nitrobenzoic acid) (DTNB, 98%), hyaluronidase (HAD, ⩾300IU/mg) and methylene blue (MB, ⩾70%) were purchased from Shanghai Aladdin Bio-Chem Technology Co., LTD. Lysotracker Green DND-26 was purchased from Invitrogen (U.S.). Cu^{2+} probe (Rhodamine B hydrazide, RBH) was purchased from Fount Beijing Bio-Tech Co., LTD. 1,2-bis (2-aminophenoxy) ethane-N, N, N', N'-tetraacetic acid tetrakis (acetoxymethyl ester) (BAPTA-AM) was purchased from Glpbio Technology (Shanghai, China). Reactive Oxygen Species Assay Kit, Hydrogen Peroxide Assay Kit, Mitochondrial Membrane Potential Assay Kit with JC-1, and Enhanced ATP Assay Kit were purchased from Beyotime Biotechnology (Shanghai, China). Fluo-8 AM was purchased from Jiangsu Keygen Biotech Corp., Ltd. CheKine Reduced Glutathione (GSH) Colorimetric Assay Kit was purchased from Abbkine Scientific Co., Ltd. (Wuhan, China). Alizarin red（Mw=342.26）was purchased from Yunaye (Shanghai, China). Cytochrome C（Cyt-C）antibody and ferredoxin 1 (FDX1) antibody were purchased from Abcam. Dihydrolipoamide S-acetyltransferase (DLAT) polyclonal antibody was purchased from Proteintech (Rosemont, USA). TNF-α ELISA Kit, IL-6 ELISA Kit, and IFN-γ ELISA Kit were purchased from

Quanzhou Jiubang Biotechnology Co., Ltd. All other reagents and materials were directly obtained from the lab and were commercially available.

Synthesis of MOF-199 (CuII). The MOF-199 was synthesized by the typical method.[1] Trimesic acid (1.25 mmol) and copper nitrate trihydrate (2.85 mmol) were separately added into a mixture of ethanol, deionized water (10 mL, v:v, 1:1). After mixing the above solutions, triethylamine (250 mL) was slowly added and stirred for 30 min at room temperature. After centrifugation, the product was washed twice with water and three times with ethanol. The final product was stored with anhydrous ethanol at room temperature.

Synthesis of ultrasmall CaO_2 NPs. The CaO_2 was prepared according to previous reports.[2] Calcium chloride (2.7 mmol), ammonia solution (1.5 mL, 1 M), and PEG400 (12 mL) were dissolved in 3 mL deionized water, then the mixture was stirred at room temperature（900 rpm, 2 h）. Then 1.5mL H_2O_2 (30%) was slowly dropwise added into the mixture. After stirring for 2 h, NaOH (0.1 M) solution was added to the solution until the pH reached 8.5. The precipitation was collected by centrifugation, washed with ethanol and filtered to obtain ultrasmall CaO_2 NPs. The final product was stored with anhydrous ethanol at room temperature.

Synthesis of HA-CD@MOF. MOF199 (2 mg) and DOX (2 mg) were added into 2 ml of anhydrous ethanol. After stirring the mixture for 12 h, CaO_2 (2 mg) was added to the above solution and kept stirring for overnight. Next, HA (3mg) was

added and stirred for another 12 h. All these procedures were carried out at room temperature. After centrifugation with anhydrous ethanol and deionized water, the final product was collected and stored at -20℃.

Characterization. The morphology of HA-CD@MOF NPs was observed by transmission electron microscopy (TEM, FEI Tecnia G2 12, USA). The zeta potentials and size distributions of HA-CD@MOF NPs, CD@MOF NPs, DOX@MOF NPs and Cu-MOF NPs were determined using the ζ potential and dynamic light scattering (DLS, Malvern, NanoZS, UK). The presence of DOX in the NPs was determined by UV–vis spectroscopy (Shimadzu, UV-3600, Japan). The spectrum of HA-CD@MOF NPs (dissolved in DMSO) and DOX solutions at different concentrations were tested and calculated for the standard concentration curve to analyze the content of DOX in HA-CD@MOF NPs. The entrapment efficiency and content of DOX were determined by Eq. (1). Drug entrapment efficiency (%) = Mass of entrapped Drug / Mass of total Drug × 100%. The existence of Ca^{2+} and Cu^{2+} in HA-CD@MOF NPs was determined by X-ray photoelectron spectra (XPS, Thermo Scientific Escalab 250Xi, USA) and the quantitative analysis of Ca^{2+} and Cu^{2+} in HA-CD@MOF NPs were determined by inductively coupled plasma-mass spectrometry (ICP-MS, Aglient 7800, Japan).

DOX and ions released from HA-CD@MOF NPs. Firstly, HA-CD@MOF NPs suspension was equivalently added into solutions with different conditions: (1) control; (2) GSH; (3) HAD; (4) GSH + HAD. Then, the above solutions were

stirred at 150 rpm at room temperature. 2 mL solution of each group was collected at different time points (0, 0.5, 1, 3, 6, 12, 24 and 48 h) for measurement by UV–vis spectroscopy and ICP-MS to calculate the cumulative release ratio of DOX, Ca^{2+} and Cu^{2+} under different conditions.

H_2O_2 production. 50 μl H_2O_2 solution (0, 6.25, 12.5, 25, 50, and 100 μM) was added to a 96-well plate, and then each well was added with 100μl of H_2O_2 Assay Buffer. After reacting at room temperature for 30 minutes, UV-vis spectra were used to measure the absorbance at 560nm, and then the standard concentration curve was calculated. 0.2 mg of HA-CD@MOF NPs with or without HAD was added into 2 mL solutions with GSH (10mM), and stirred for 1, 6, and 12 h, respectively. After centrifugation, 50 μl supernatants of each group were added to a 96-well plate and mixed with H_2O_2 Assay Buffer. Then, the intracellular level of H_2O_2 was calculated.

GSH depletion detection *in vitro*. GSH responsiveness of HA-CD@MOF NPs was studied by DTNB. Firstly, the absorption of GSH (0, 0.625, 1.25, 2.5, 5, and 10 mM) was detected at 412 nm and the standard concentration curve was calculated. Then, 180 μl HA-CD@MOF NPs with or without HAD were mixed with 20 μl GSH. After reacting for 6h, the supernatants were collected and mixed with DTNB (3mg/ml) for 30 min. The absorption at 412 nm was detected by UV−vis spectra and the concentration of GSH in supernatant was calculated.

·OH production detection *in vitro*. ·OH production of NPs was studied by MB. First, GSH (10 mM) and 100 μg/ml of different NPs: (1) Cu-MOF NPs; (2) DOX@MOF NPs; (3) CD@MOF NPs; (4) HA-CD@MOF NPs, were mixed and then reacted for 30 min. Then, H_2O_2 (10 mM) was added and reacted for an extra hour. After that, MB (25 μg/ml) was added and reacted for 30 min. At last, the MB degradation mediated by ·OH was monitored by UV−vis spectra from 420-700nm. The supernatants were also collected and detected by ESR spectra.

Cell culture and tumor-bearing animal model establishment. 4T1 cells and HUVECs were cultured in RPMI-1640 medium containing 10% fetal bovine serum and 100 U/mL penicillin-streptomycin solution. The cells were maintained in a 5% CO_2 incubator at 37°C. Female BALB/c mice, weighing approximately 20 g and aged 6-8 weeks, were housed in a humid environment, and food and water were free to access. All animal experiments were conducted according to the guidelines and regulations approved by the Ethics Committee of the Second Affiliated Hospital of Chongqing Medical University. To establish the artificial tumor model, each BALB/c mouse was injected with 1×10^6 4T1 cells suspended in 100 μL PBS solution into the right breast fat pad. The tumor volume was calculated using the formula [$\pi/6 \times$ length $\times$ (width)^2].

Cellular uptake and distribution *in vitro*. 4T1 cells and HUVECs were seeded into confocal cell culture dishes and were treated with HA-CD@MOF NPs for different durations (0.5, 1, 2, and 4 h). After fixing these cells with

paraformaldehyde (4%), the cell nuclei were stained with DAPI. CLSM (Nikon A1 instrument, Japan) and flow cytometry (Becton Dickinson FACS Vantage instrument, USA) were invited to analyze the uptake of HA-CD@MOF NPs by 4T1 cells and HUVECs. To detect the lysosome inoculation, the cells were incubated with HA-CD@MOF NPs (60 μg/mL) or DOX (10 μg/mL) for 2 h. Then, the cell medium was removed and Lysotracker Green DND-26 was added to incubate with the cells for 15 min. The subcellular localization of HA-CD@MOF NPs was evaluated using CLSM. The cells incubated with HA-CD@MOF NPs for 1, 2, 6, and 12h were also collected and observed under Bio-TEM.

Cytotoxicity *in vitro*. To investigate the biocompatibility of HA-CD@MOF NPs, HUVECs were cultured in 96-well plates with HA-CD@MOF NPs (0, 20, 40, 60, 80, 100, 120, and 140 μg/mL) for 12 h. Cell viability was then determined using a CCK-8 assay. For evaluating the anti-cancer efficacy of HA-CD@MOF NPs, 4T1 cells were subjected to different treatments (n = 3): (1) Control; (2) Cu-MOF NPs; (3) DOX@MOF NPs; (4) CD@MOF NPs; (5) HA-CD@MOF NPs for 6 hours. Cell viability was assessed using the CCK-8 assay. Additionally, cellular apoptosis was also assessed through flow cytometry and CLSM.

H_2O_2 production of 4T1 cells *in vitro*. 4T1 cells were seeded into tissue culture dishes and cultured for 6 hours with (1) Control; (2) Cu-MOF NPs ; (3) DOX@MOF NPs; (4) CD@MOF NPs; (5) HA-CD@MOF NPs. The cells were then collected and given 200ul of Lysis Buffer to be fully lysed. After that, the

supernatants were collected by centrifuge at 12000×g at 4℃ for 3-5 minutes. Next, 50 μl supernates of each group were added to a 96-well plate and mixed with H_2O_2 assay Buffer. The absorbances of the mixtures at 540 nm were measured and the H_2O_2 concentration of each group were calculated.

ROS detection of 4T1 cells *in vitro*. 4T1 cells were seeded into laser confocal cell culture dishes and treated differently for 6 hours: (1) Control; (2) Cu-MOF NPs; (3) DOX@MOF NPs; (4) CD@MOF NPs; (5) HA-CD@MOF NPs; (6) HA-CD@MOF NPs + BATPA-AM. After treatment, the DCFH-DA probe was added and incubated for 30 minutes. The cells were then washed three times with PBS and fixed with 4% paraformaldehyde. Intracellular ROS levels were evaluated using CLSM and flow cytometry.

GSH consumption evaluation of 4T1 cells *in vitro*. 4T1 cells were seeded into 6-well plates and were treated differently for 6 h: (1) Control; (2) Cu-MOF NPs ; (3) DOX@MOF NPs; (4) CD@MOF NPs; (5) HA-CD@MOF NPs. Then, the intracellular GSH levels were examined by the CheKine Reduced Glutathione (GSH) Colorimetric Assay Kit.

Intracellular Ca^{2+} level of 4T1 cells. 4T1 cells were seeded into confocal cell culture dishes and were treated differently for 6 hours: (1) Control; (2) Cu-MOF NPs ; (3) DOX@MOF NPs; (4) CD@MOF NPs; (5) HA-CD@MOF NPs. After that, the Fluo-8 AM probe was introduced and allowed to incubate for 30 minutes.

After washing the cells three times with PBS, intracellular Ca^{2+} levels were assessed both visually and quantitatively using CLMS and flow cytometry.

Cell calcification detection. To examine the calcification ability of HA-CD@MOF NPs, 4T1 cells were seeded into 12-well plates and incubated with HA-CD@MOF NPs (60 μg/mL, 1 mL) for different durations (3, 6, 12, and 24 hours). Subsequently, the cells were collected and subjected to overnight fixation using 95% ethanol. 2 mL of alizarin red dispersed in tris buffer (0.1%, pH = 8.5) were added and incubated for 30 minutes. Then the cells were washed three times with PBS and examined with a light microscope.

Intracellular Cyt-C level of 4T1 cells. 4T1 cells were seeded into confocal cell culture dishes and were treated differently for 6 h: (1) Control; (2) Cu-MOF NPs ; (3) DOX@MOF NPs; (4) CD@MOF NPs; (5) HA-CD@MOF NPs. After a fixation by 4% paraformaldehyde, a 40-min blockage using 5% BSA was performed. The cells were then incubated overnight at 4 ℃ with rabbit anti-Rabbit Cytochrome C（Cyt-C）primary antibodies. Subsequently, FITC-labeled goat anti-rabbit secondary antibodies were applied in the dark for 1 hour. Finally, the nuclei were stained with DAPI, and the Cyt-C expression level within 4T1 cells was measured by CLSM.

Mitochondria integrity assay *in vitro*. 4T1 cells were seeded into confocal cell culture dishes and were subjected to various treatments for 6 hours: (1) Control; (2) Cu-MOF NPs ; (3) DOX@MOF NPs; (4) CD@MOF NPs; (5) HA-CD@MOF

NPs. After removing the medium, JC-1 dye was introduced and incubated for 30 minutes at 37 ℃. After washing the cells three times with PBS， the mitochondrial membrane potential (MMP) was investigated by CLMS. Additionally, the mitochondrial morphology of 4T1 cells, both with and without HA-CD@MOF NPs treatment, was examined using cryo-transmission electron microscopy.

ATP reduction evaluation of 4T1 cells. 4T1 cells were seeded into 6-well plates and were subjected to different treatments for 6 hours: (1) Control; (2) Cu-MOF NPs ; (3) DOX@MOF NPs; (4) CD@MOF NPs; (5) HA-CD@MOF NPs. After that, the medium was removed and the intracellular level of ATP was examined using the Enhanced ATP Assay Kit by a luminometer.

Cu-ATPase activity detection of 4T1 cells. 4T1 cells were seeded into tissue culture dishes and cultured for 6 hours with: (1) Control; (2) Cu-MOF NPs; (3) HA-CD@MOF NPs. Then the cells were washed and collected for Cu-ATPase activity measurement using the Cu-ATPase assay kit.

Intracellular Cu^{2+} concentration measurement of 4T1 Cells. 4T1 cells were seeded into tissue culture dishes and cultured for 6 hours with: (1) Control; (2) Cu-MOF NPs; (3) HA-CD@MOF NPs. Then the cells were washed and collected for ICP-MS (Perkin Elmer, USA). 4T1 cells were also seeded into confocal cell culture dishes and given the treatments above. Then the cells were washed and

collected. After incubation with the Cu^{2+} probe for 30 min, the cells were immediately observed using CLSM.

Cuproptosis-related protein evaluation *in vitro*. 4T1 cells were seeded into tissue culture dishes and cultured for 6 hours with: (1) Control; (2) Cu-MOF NPs; (3) HA-CD@MOF NPs. The cells were then washed and collected for western blotting to detect the expression of *FDX1*. Furthermore, the cells were seeded into confocal cell culture dishes and given the same treatments. After washing and a fixation with 4% paraformaldehyde, a 40-min blockage was performed using 5% BSA. The cells were then incubated overnight at 4 °C with *DLAT* antibody and followed by an incubation with FITC-labeled goat anti-rabbit secondary antibody in the dark for an additional hour. After staining the nuclei with DAPI, the expression levels of *DLAT* aggregation in 4T1 cells were examined using CLSM.

Anticancer effect of HA-CD@MOF NPs *in vivo*. 25 female tumor-bearing BALB/c mice were randomly divided into five groups (each group consists of five mice, n = 5) and treated as follows: (1) Control; (2) Cu-MOF NPs; (3) DOX@MOF NPs; (4) CD@MOF NPs; (5) HA-CD@MOF NPs. Intravenous treatments were administered every 4 days, with each mouse receiving the corresponding treatment at a dose of 2.8 mg/kg in a volume of 0.1 mL. The control group received a saline solution instead. Body weights and tumor volumes of the mice were measured every 2 days throughout the study. After 2 weeks, all mice were sacrificed, and their tumors were collected for further analysis. The tumor

inhibition rates of each group were calculated based on the following equation: tumor inhibition rates = ($V/W_{14,\ control}$ – $V/W_{14,\ experiment}$)/$V/W_{14,\ control}$ × 100%, where $V/W_{14,\ control}$ and $V/W_{14,\ experiment}$ refers to the tumor volume or tumor weight of mice in Control group and experiment groups on the day 14, respectively. Hematoxylin-eosin (H&E) staining, as well as immunohistochemical analysis of proliferating cell nuclear antigen (PCNA) and TdT-mediated dUTP nick-end labeling (TUNEL) of tumors were conducted. The ROS level in the tumor tissues of the different groups was also assessed using immunohistochemical analysis. Additionally, the heart, liver, spleen, lung, and kidney of the mice were excised and subjected to H&E staining.

Detection of ICD induced by HA-CD@MOF NPs. To detect the ICD biomarkers, 4T1 cells were cultured in confocal cell cultures and were subjected to different treatments for 6 hours: (1) Control; (2) Cu-MOF NPs ; (3) DOX@MOF NPs; (4) CD@MOF NPs; (5) HA-CD@MOF NPs. After removing the medium, the cells were washed. After a fixation with 4% paraformaldehyde, a 40-min blockage using 5% BSA was performed. The cells were then incubated overnight at 4 °C with rabbit anti-Rabbit chaperone calreticulin (CRT) or large amounts of high-mobility group box 1 (HMGB1) primary antibodies. FITC-labeled goat anti-rabbit secondary antibodies were then applied in the dark for an additional hour. After staining the nuclei with DAPI, the expression levels of CRT and HMGB1 in 4T1 cells were examined using CLSM. To detect the dendritic cells (DCs) maturation level induced by HA-CD@MOF NPs. Immature DCs were

co-cultured with 4T1 cells in a transwell system. Then flow cytometry was invited to evaluate the ratio of CD80+ CD86+ cells using CD45+ CD11b+ CD11c+ as a gate. In vivo, the mice were also given the above treatments. After removing the tumor, immunohistochemical analysis of CRT was performed to detect the ICD Biomarkers.

Detection of metastasis suppression ability of HA-CD@MOF NPs *in vitro*. Cell invasion and migration were assessed to evaluate the metastasis suppression ability of HA-CD@MOF NPs in vitro. Firstly, a transwell system was established to perform migration and invasion assays respectively. 4T1 cells after different treatments were then suspended in a serum-free medium and added into the upper chamber, while the lower chamber was added with 20% FBS medium. After a 24-hour incubation, the migrated or invaded cells were fixed with 100% methanol and stained with 1% crystal violet. Finally, cells were imaged and counted under a microscope. Besides, scratch wound assay was also performed to investigate the migration of 4T1 cells. 4T1 cells were first cultured into 6-well plates and treated differently. After creating the scratch wound, cells were washed with PBS and then incubated with serum-free culture medium for 24 hours. Photographs at various time points (0 and 24h) were taken randomly and further analyzed using image J.

Detection of metastasis suppression ability of HA-CD@MOF NPs *in vivo*. 25 female tumor-bearing BALB/c mice were randomly divided into five groups (n =

5) and each group received different treatments every 4 days : (1) Control; (2) Cu-MOF; (3) DOX@MOF; (4) CD@MOF (5) HA-CD@MOF. After 8 days, 4T1 cells were then injected intravenously to build a lung-metastasis model. On the 22nd day, and the lungs were collected after the mice were sacrificed. The lungs were then stained by rinitrophenol and H&E.

PAI/MRI imaging ability of HA-CD@MOF NPs *in vivo*. 4T1 tumor-bearing mice were injected with HA-CD@MOF NPs and CD@MOF NPs solutions, respectively, n=3. Images were recorded at various time points (pre-injection and, 1, 3, 6, 12, and 24 h post-injection), using a photoacoustic imaging system (Vevo LAZR, Canada) and magnetic resonance imaging system (3.0T, Siemens).

Fluorescence imaging (FLI) and biodistribution *in vivo*. 4T1 tumor-bearing mice were injected with DIR-labeled HA-CD@MOF and CD@MOF solution intravenously, respectively. FLI was acquired at pre-injection and, 2, 4, 6, 8 24 48 and 72h post-injection via fluorescence imaging system (Cri Inc, USA), the relative FL intensity of the tumor and the main organs (Heart, Liver, Spleen, Lung, and Kidney) were also recorded in ex vivo experiment after the mice were sacrificed.

Biosafety assay. 30 BALB/c mice were randomly divided into five groups (n = 5) and sacrificed after receiving intravenous injections of 200 μL HA-CD@MOF (2 mg/mL) for different time points (0, 1, 3, 7, 14, and 28 days). The blood of

each mouse was collected for routine blood examination and biochemistry assay. The major organs of the mice were also collected and stained with H&E.

Statistical analysis. All data were subjected to statistical analysis using GraphPad Prism 9.0 software. The data were presented as means ± SD (standard deviation). One-way and two-way analysis of variance (ANOVA) tests and Student's t-test were performed to evaluate the statistical significance of the data. Significance was considered at $^{*}p < 0.05$, $^{**}p < 0.01$, and $^{***}p < 0.001$.

Supplementary Figures

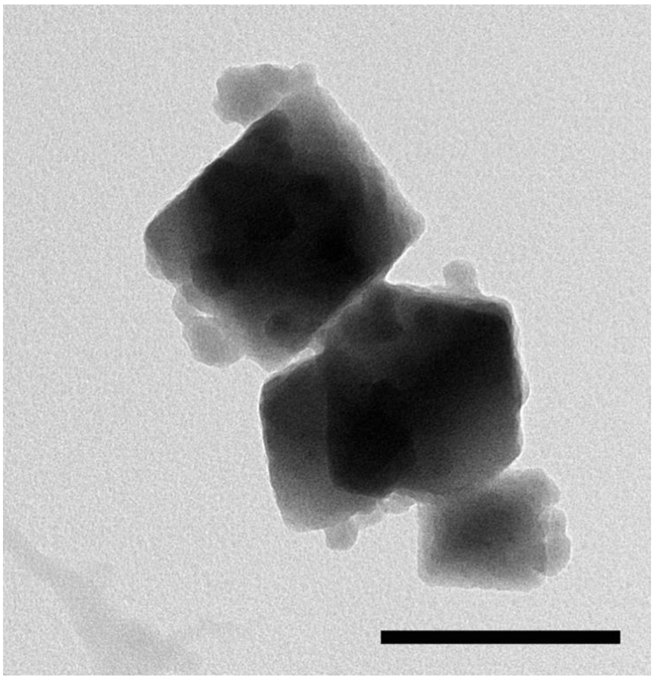

Figure S1. TEM image of MOF-199 NPs, the scale bar is 200 nm.

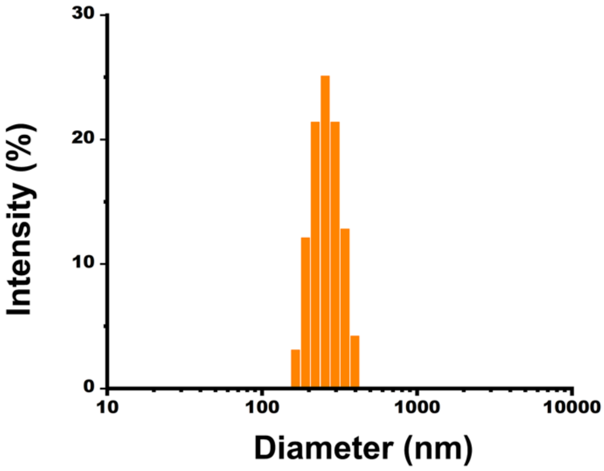

Figure S2. Size distribution of HA-CD@MOF NPs.

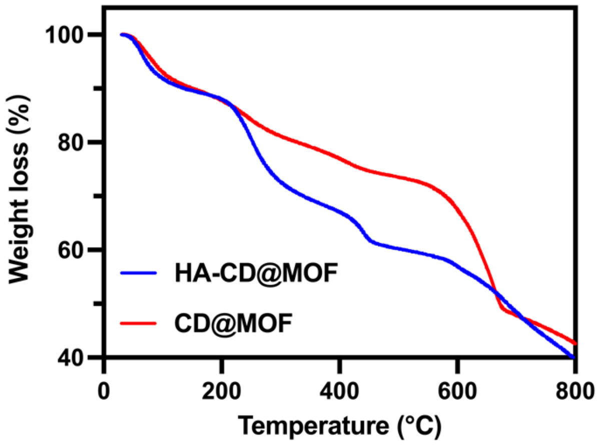

Figure S3. TGA curves of HA-CD@MOF NPs and CD@MOF NPs over the temperature range from 25 to 800 °C.

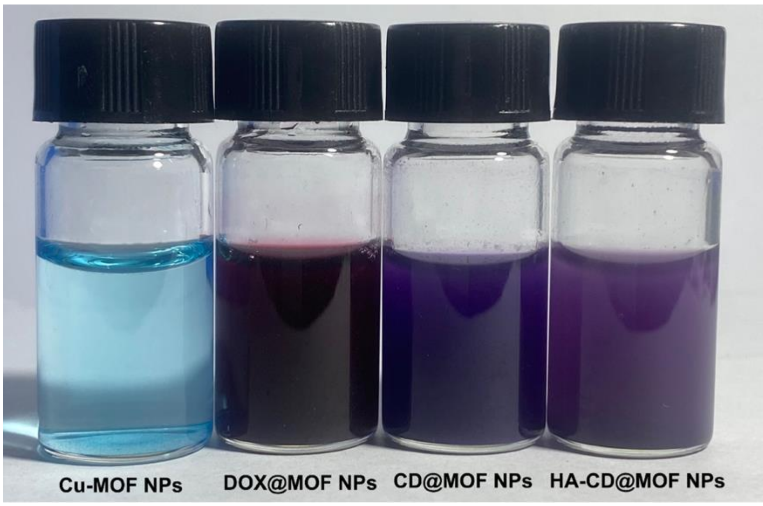

Figure S4. Digital photography of Cu-MOF NPs, DOX@MOF NPs, CD@MOF NPs and HA-CD@MOF NPs.

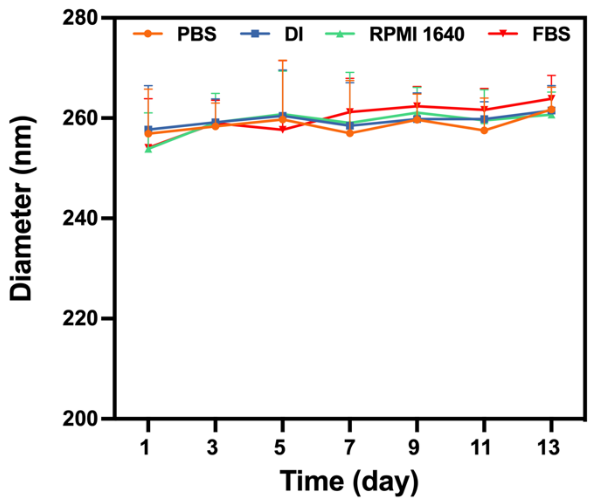

Figure S5. Size distribution of HA-CD@MOF NPs dispersed in various physiological solutions for 13 days, n = 3. Results are presented as means ± SD.

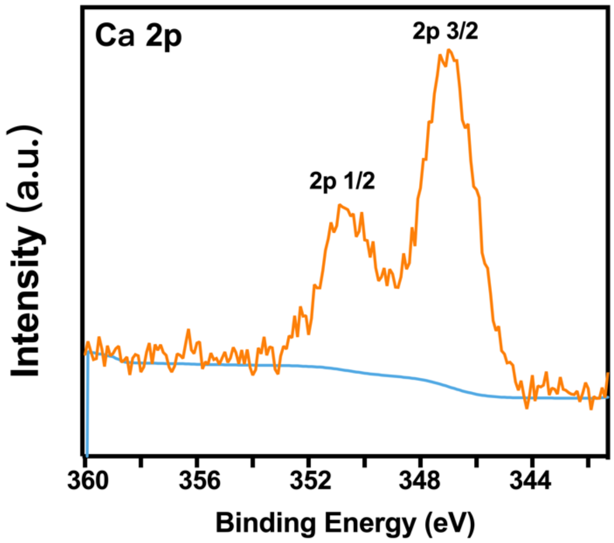

Figure S6. Cu 2p XPS spectrum of HA-CD@MOF NPs.

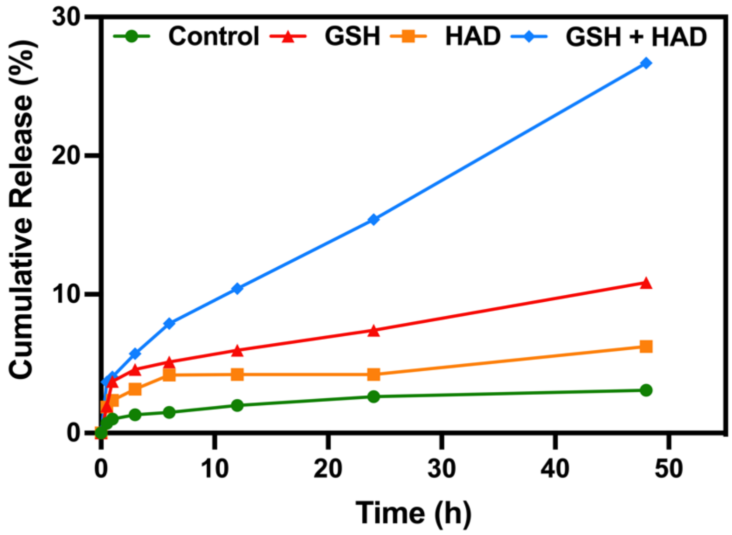

Figure S7. Cu^{2+} release from HA-CD@MOF NPs under different conditions and time intervals.

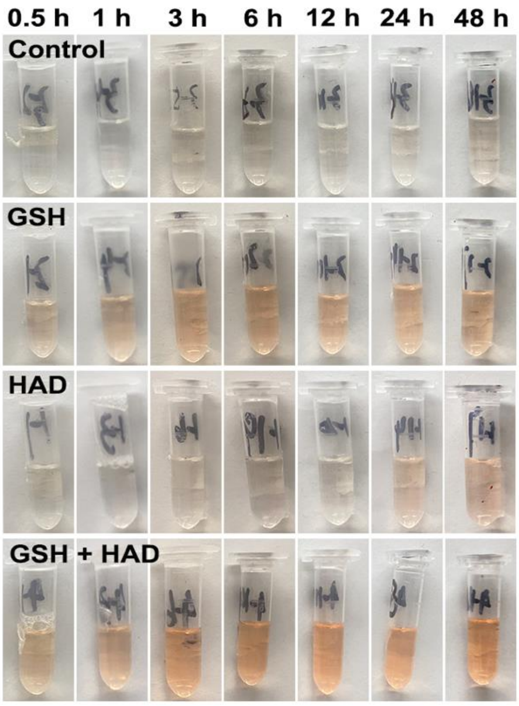

Figure S8. The color changes of the buffers with HA-CD@MOF NPs under different conditions at different time points, the orange color refers to the released DOX

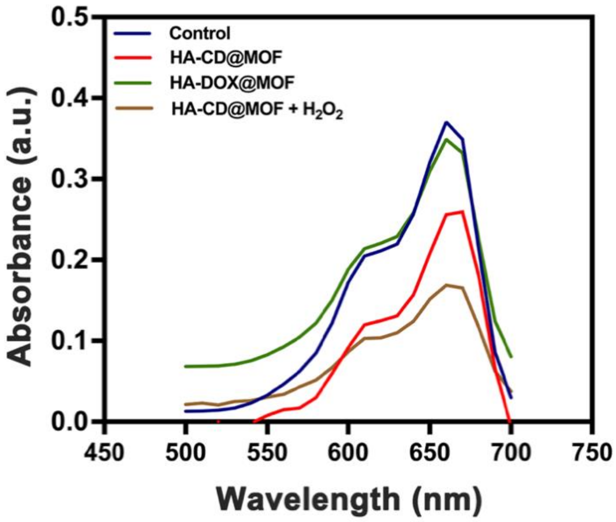

Figure S9. The MB degradation rates under different conditions, all groups were incubated with GSH (10mM) and HAD (100 U/mL).

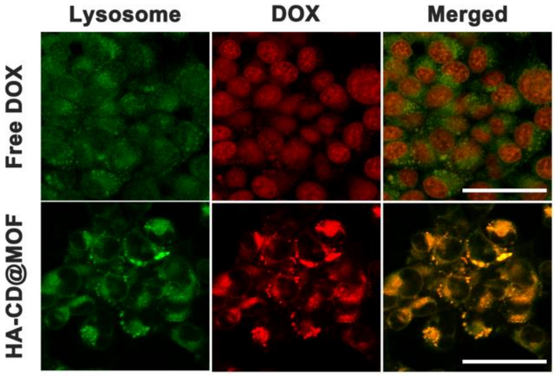

Figure S10. CLSM images of 4T1 cells after treatment with free DOX and HA-CD@MOF for 2 h, showing the intracellular location of free DOX and HA-CD@MOF NPs, respectively. The red fluorescence refers to free DOX and HA-CD@MOF NPs, and the green fluorescence refers to lysosomes, the scale bars are 50 μm.

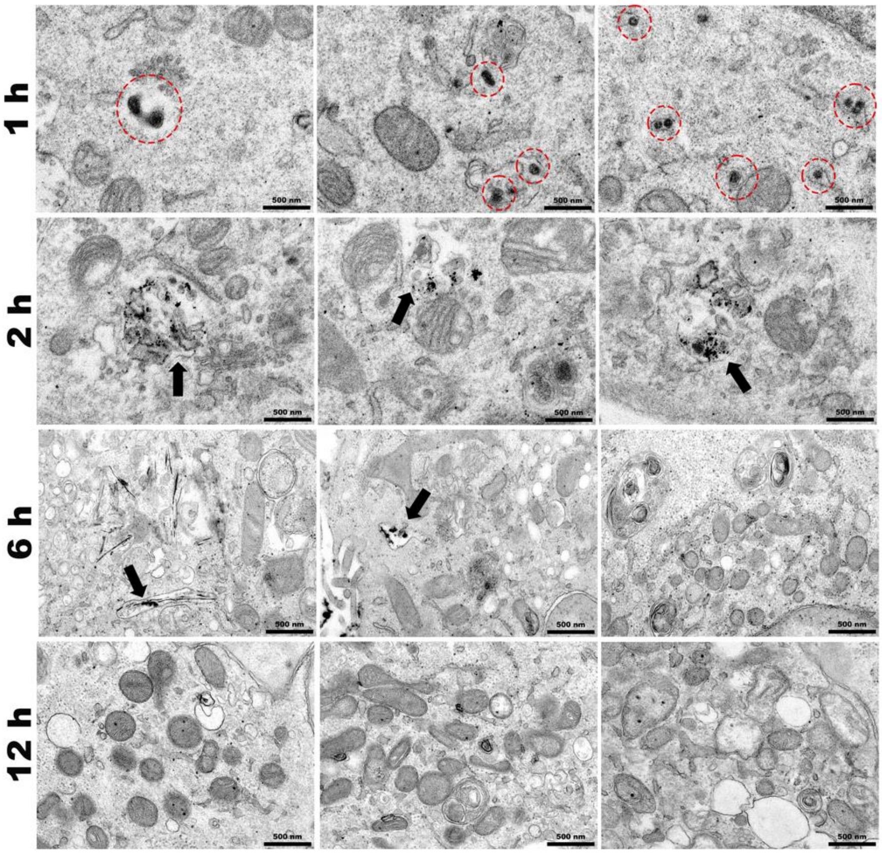

Figure S11. Bio-TEM images of 4T1 cells treated by HA-CD@MOF NPs for 1, 2, 6, and 12h, respectively. The red circles represent the HA-CD@MOF NPs endocytosed by 4T1 cells, the black arrows represent the degraded HA-CD@MOF NPs.

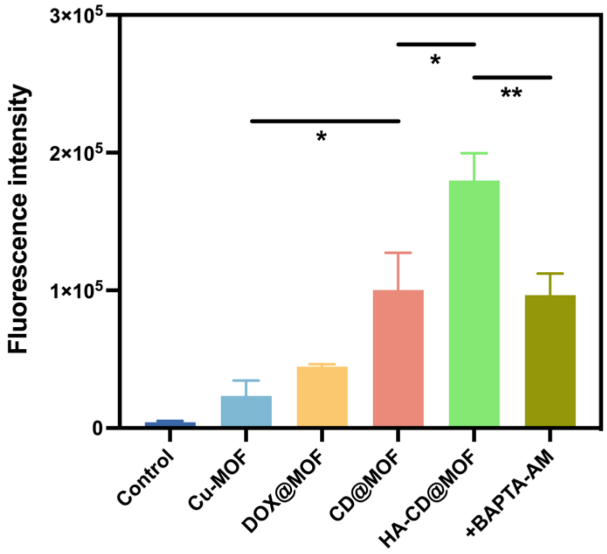

Figure S12. Quantitative analysis of intracellular ROS levels evaluated by flow cytometry after various treatments, n = 3. Results are presented as means ± SD. *P < 0.05, **P < 0.01.

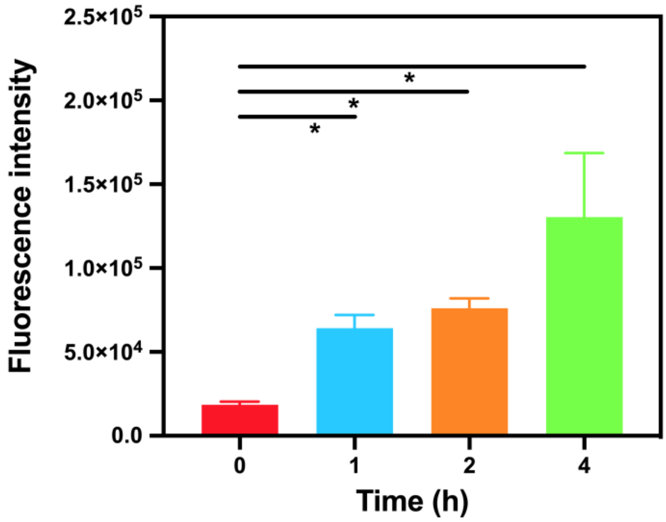

Figure S13. Quantitative analysis of intracellular Ca^{2+} levels evaluated by flow cytometry after various treatments. Results are presented as means ± SD, n = 3. *P < 0.05.

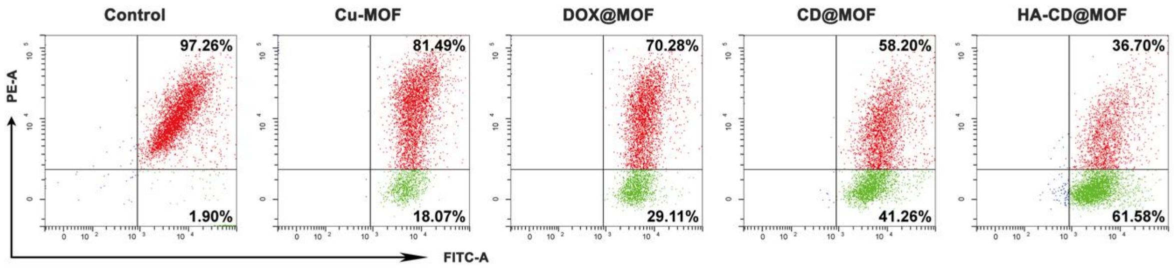

Figure S14. Flow cytometry analysis of JC-1 stained tumor cells after different treatments.

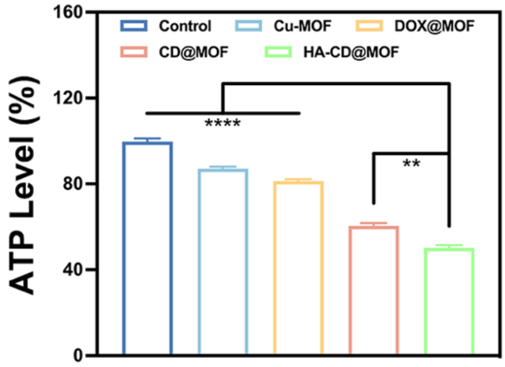

Figure S15. ATP levels in 4T1 cells after different treatments; n =3. Results are presented as means ± SD. *P < 0.05.

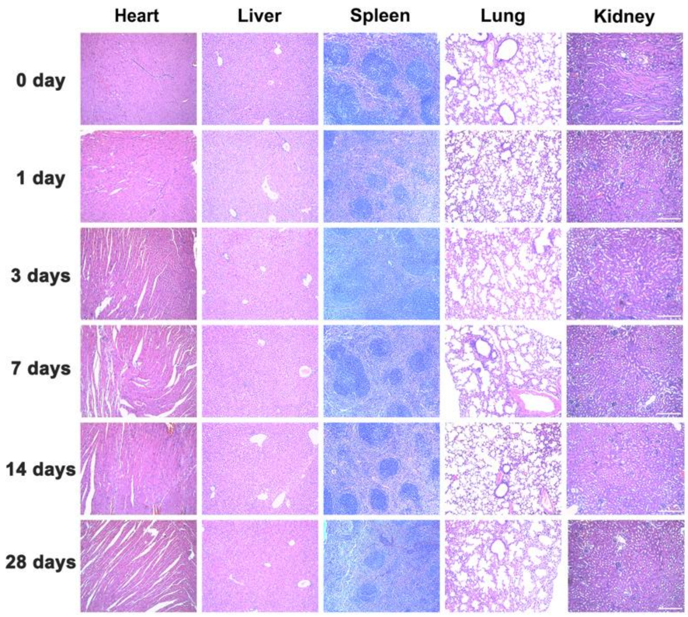

Figure S16. H&E staining of the main organs (heart, liver, spleen, lung, and kidney) in mice sacrificed at different time intervals (0 day, 1 day, 3 days, 7 days, 14 days, 28 days) after intravenous injection of HA-CD@MOF NPs, the scale bars are 500 μm.

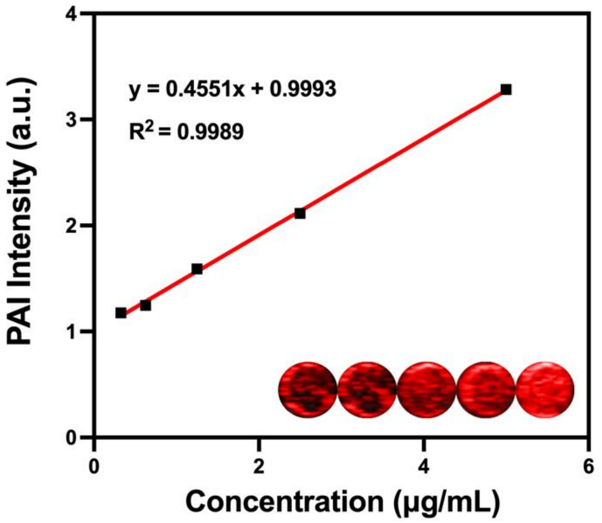

Figure S17. PAI values and PAI images of HA-CD@MOF NPs at different concentrations *in vitro*.

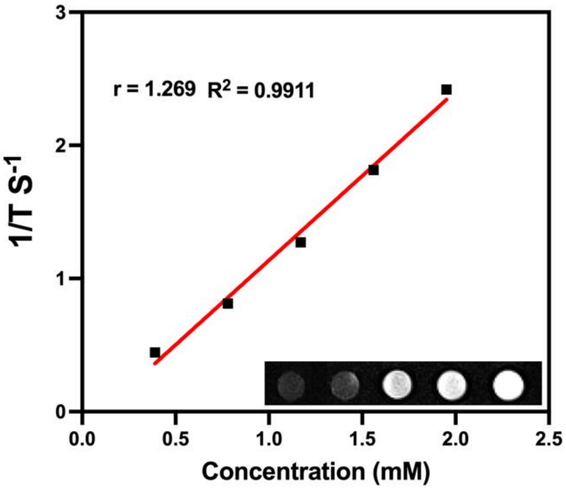

Figure S18. T1-weighted MRI images and the relaxation rate of HA-CD@MOF NPs with different concentrations *in vitro*.

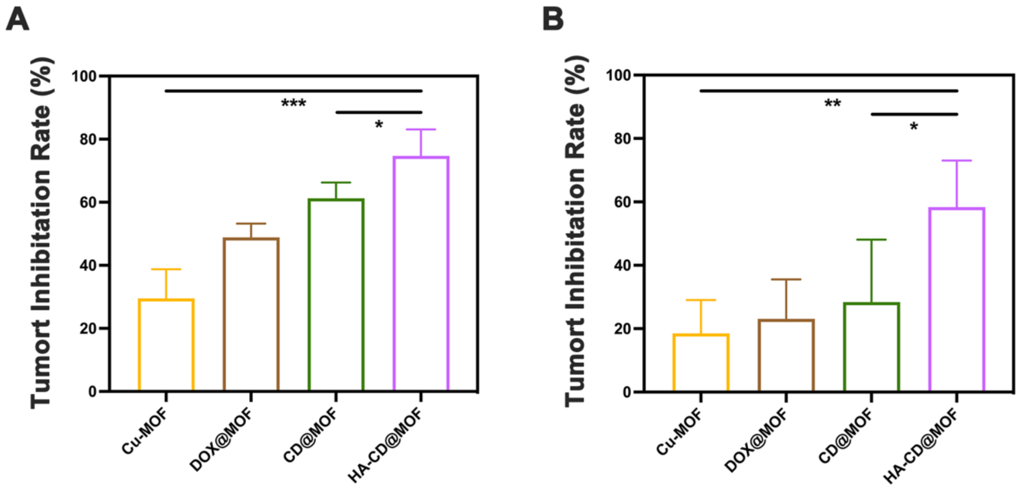

Figure S19. Tumor inhibition rates of treatment groups as compared to the control group, were calculated based on both tumor volumes (**A**) and tumor weights (**B**), n = 5. Results are presented as means ± SD. *P < 0.05.

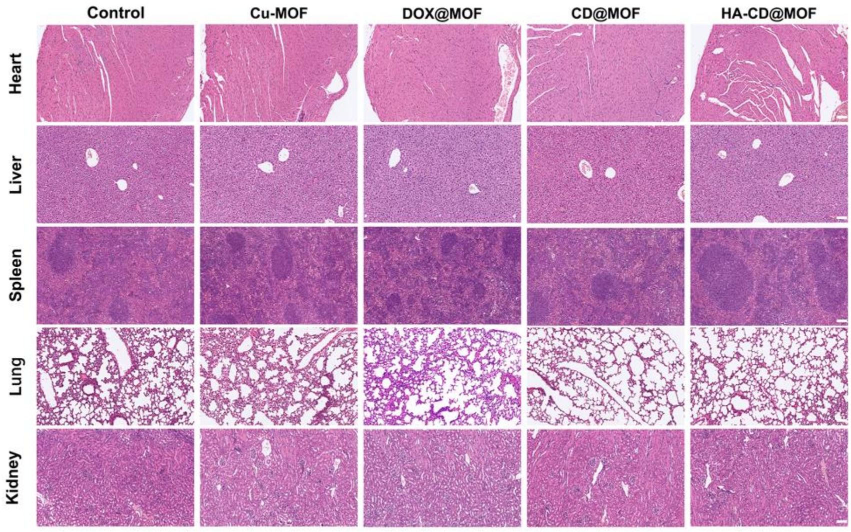

Figure S20. H&E staining of the major organs in mice sacrificed after 14 days of various treatments, the scale bars are 100 μm.

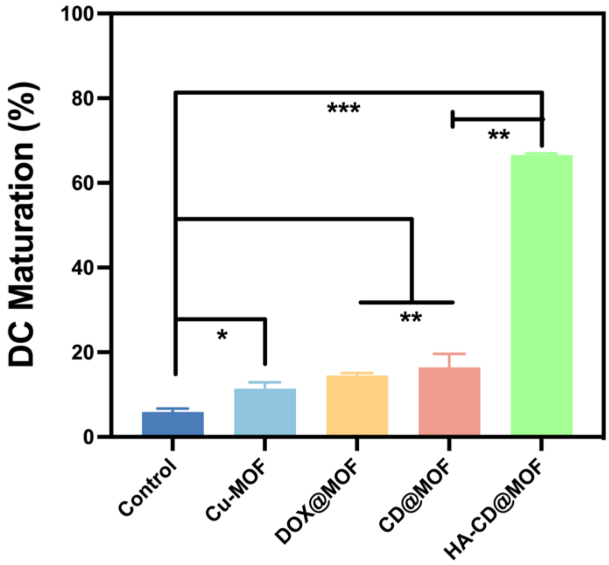

Figure S21. Quantitative analysis of DCs maturation levels after different treatments *in vitro*, n = 3. Results are presented as means ± SD. *$p < 0.05$, **$p < 0.01$ and ***$p < 0.001$.

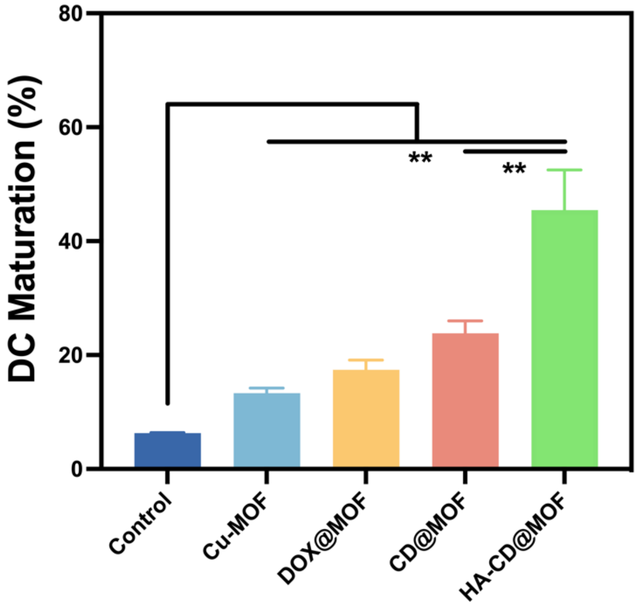

Figure S22. Quantitative analysis of DCs maturation levels after different treatments *in vivo*, n = 3. Results are presented as means ± SD. *p < 0.05, **p < 0.01.

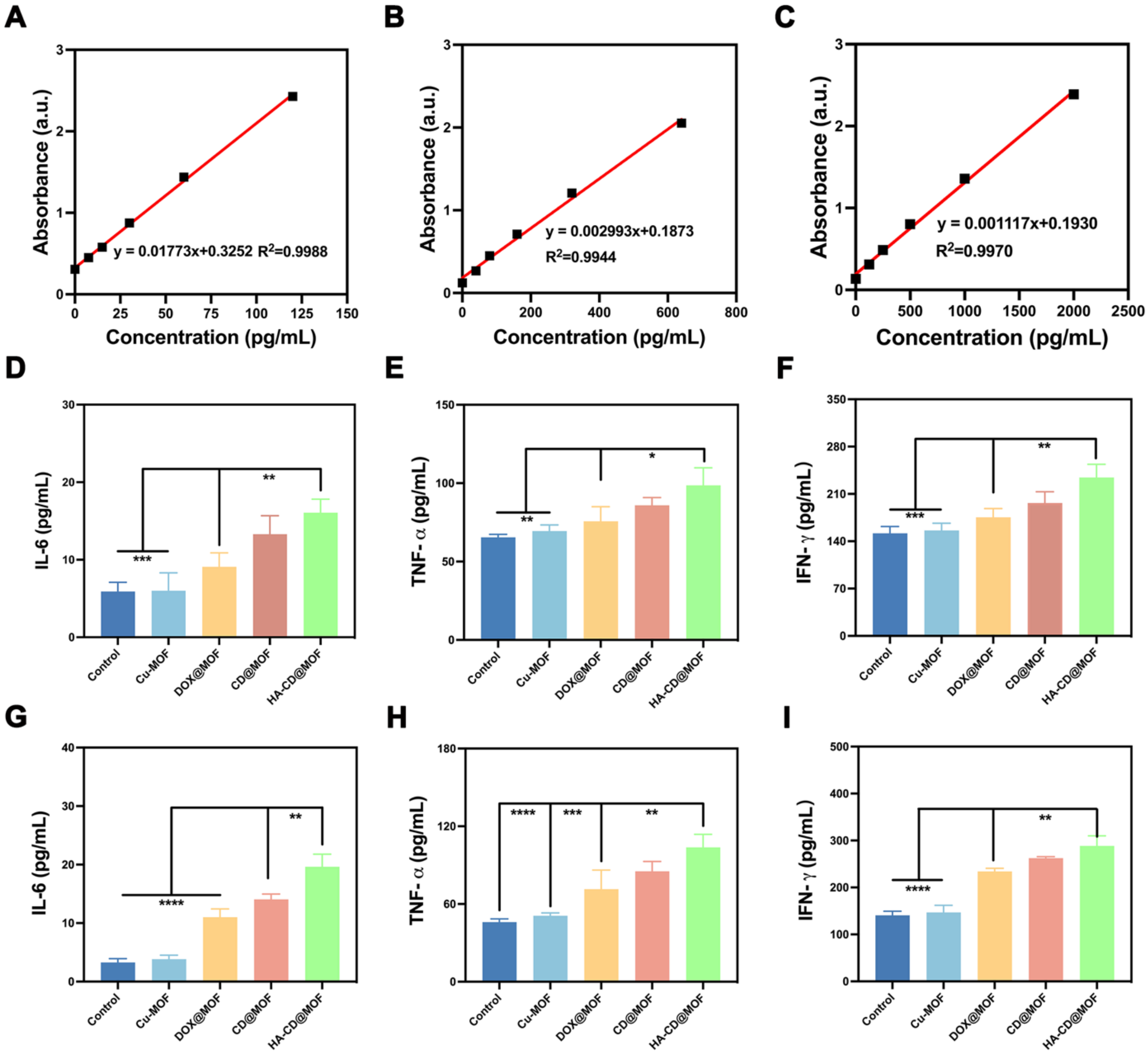

Figure S23. ELISA Cytokine (IL-6, TNF-α, IFN-γ) levels after treatment in vitro (the second row, D-E) and in vivo (the third row, G-I), n = 5. Results are presented as means ± SD. *P < 0.05, **P < 0.01.

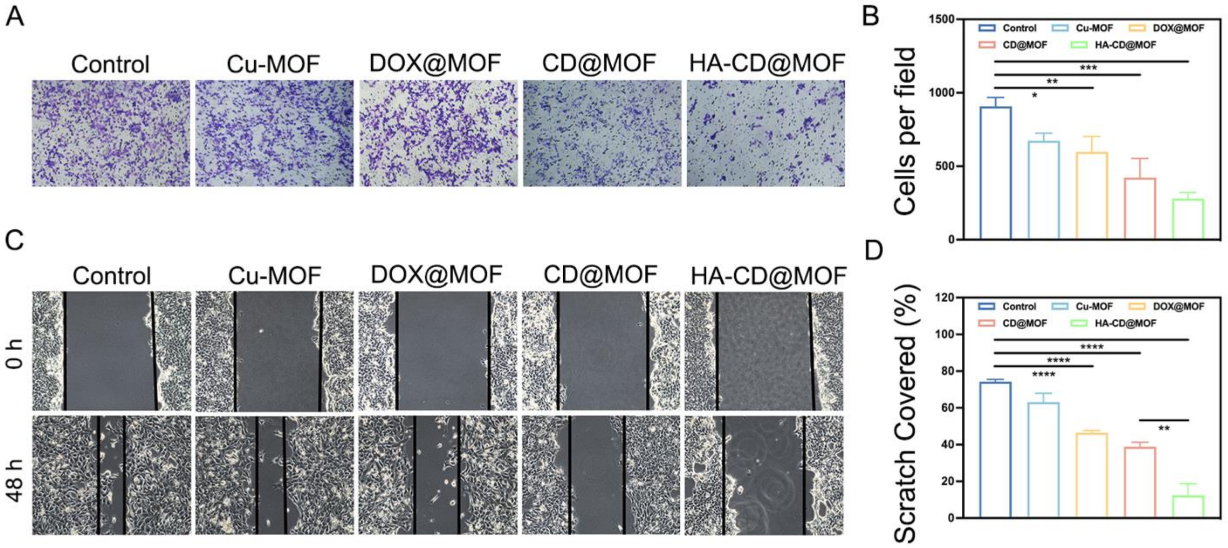

Figure S24. Cell migration ability assessed by transwell chamber assays after various treatments (A, B), n = 3. Cell migration ability assessed by wound healing assay after various treatments (C, D), n = 3. Results are presented as means ± SD. *P < 0.05, **P < 0.01.

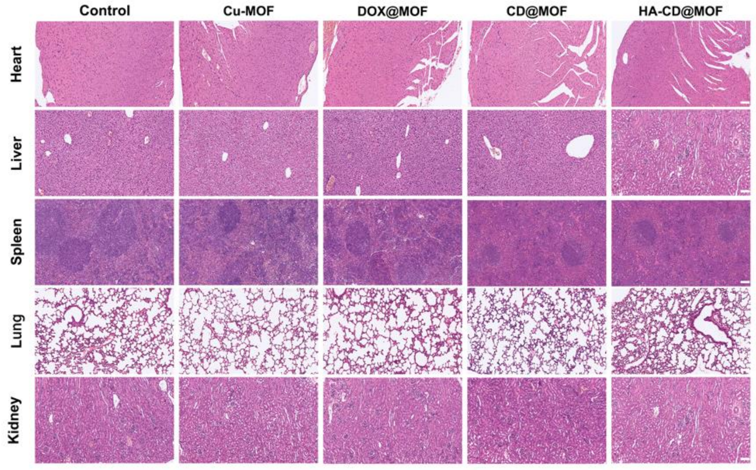

Figure S25. H&E staining of the major organs collected from mice of in vivo DC maturation analysis, the scale bars are 100 μm.

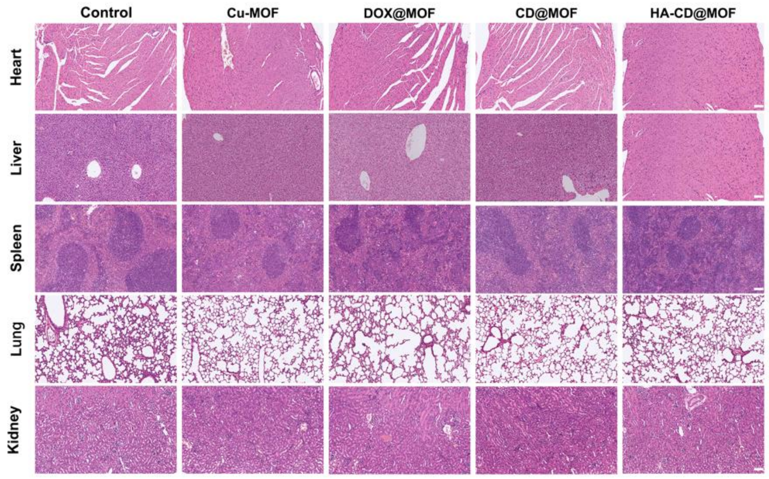

Figure S26. H&E staining of the major organs collected from mice with lung metastasis model, the scale bars are 100 μm.

Reference

[1] Y. Wang，W. Wu，J. Liu，P.N. Manghnani，F. Hu，D. Ma，C. Teh，B. Wang，B. Liu, *ACS Nano* **2019,** *13* (6), 6879-6890.

[2] C. Liu，Y. Cao，Y. Cheng，D. Wang，T. Xu，L. Su，X. Zhang，H. Dong, *Nat Commun* **2020,** *11* (1), 1735.
